# Horizon scanning of potential environmental applications of terrestrial animals, fish, algae and microorganisms produced by genetic modification, including the use of new genomic techniques

**DOI:** 10.3389/fgeed.2024.1376927

**Published:** 2024-06-13

**Authors:** Marianne Miklau, Sarah-Joe Burn, Michael Eckerstorfer, Marion Dolezel, Anita Greiter, Andreas Heissenberger, Stefan Hörtenhuber, Werner Zollitsch, Kristin Hagen

**Affiliations:** ^1^ Department of Landuse and Biosafety, Environment Agency Austria, Vienna, Austria; ^2^ Department of Sustainable Agricultural Systems, University of Natural Resources and Life Sciences, Vienna, Austria; ^3^ Federal Agency for Nature Conservation, Division Assessment Synthetic Biology/Enforcement Genetic Engineering Act, Bonn, Germany

**Keywords:** genetic engineering, genome editing, new genomic techniques, genetically modified organism, terrestrial animal, fish, algae, microorganism

## Abstract

With scientific progress and the development of new genomic techniques (NGTs), the spectrum of organisms modified for various purposes is rapidly expanding and includes a wide range of taxonomic groups. An improved understanding of which newly developed products may be introduced into the market and released into the environment in the near and more distant future is of particular interest for policymakers, regulatory authorities, and risk assessors. To address this information need, we conducted a horizon scanning (HS) of potential environmental applications in four groups of organisms: terrestrial animals (excluding insects and applications with gene drives), fish, algae and microorganisms. We applied a formal scoping review methodology comprising a structured search of the scientific literature followed by eligibility screening, complemented by a survey of grey literature, and regulatory websites and databases. In all four groups of organisms we identified a broad range of potential applications in stages of basic as well as advanced research, and a limited number of applications which are on, or ready to be placed on, the market. Research on GM animals including fish is focused on farmed animals and primarily targets traits which increase performance, influence reproduction, or convey resistance against diseases. GM algae identified in the HS were all unicellular, with more than half of the articles concerning biofuel production. GM algae applications for use in the environment include biocontrol and bioremediation, which are also the main applications identified for GM microorganisms. From a risk assessor’s perspective these potential applications entail a multitude of possible pathways to harm. The current limited level of experience and limited amount of available scientific information could constitute a significant challenge in the near future, for which risk assessors and competent authorities urgently need to prepare.

## 1 Introduction

In recent years, new genomic techniques (NGTs), also referred to as genome editing or targeted mutagenesis, for the development of genetically modified organisms (GMOs) have attracted significant attention not only in scientific research, but also in public at large. The reason is that NGTs, in particular CRISPR based methods, can be easily applied to an increasing number of organisms ranging from plants to animals and microorganisms. Moreover, they substantially expand the spectrum of possible genetic modifications in these organisms, including directed and undirected genetic or epigenetic modifications at specific genomic target sites (EC, 2017; NAS, 2017). In addition to modifying single target genes, NGTs have been developed which allow the simultaneous or successive modification of several genes at different genomic sites, e.g., by multiplexing approaches (Broothaerts et al., 2021). If multiple changes are introduced by NGTs, the resulting organisms may be considered a product of synthetic biology by the European Foods Safety Authority (EFSA) (More et al., 2020; Naegeli et al., 2021; Mullins et al., 2022). Unlike several other countries, in Europe products of NGTs are considered GMOs (CJEU 2018; CJEU 2023; see also Spranger (2023a) for a legal analysis). Thus, in accordance with this ruling of the European Court of Justice, this review covers all types of modified organisms and the term GMO in this article is used for all, regardless of the molecular biology techniques used to achieve the respective modification.

Many experts and policymakers suggest that products developed by NGTs may contribute to sustainability in various ways, e.g., by enhancing soil fertility, producing biofuels with GM microalgae, control of disease agents and vectors for pathogens, or for bioremediation (OECD, 2015). In addition, they raise expectations that the application of NGTs, particularly but not exclusively in plants, will allow or accelerate the development of organisms for use in agriculture to address an increasing demand for food and feed or to mitigate adverse effects of climate change (EC, 2021; EC, 2023a; EC, 2023b; EC, 2023c). Similar hopes have been expressed for genetically modified (GM) animals (Tizard et al., 2016). However, with respect to GM crops, according to Hüdig et al. (2022), to date there is little scientific evidence on the extent to which genome edited crops will realistically express traits that contribute to sustainability. This is partly due to the fact that such traits are complex, context dependent and at present not well defined (e.g., drought tolerance). Traits that could contribute to sustainability are not abundant among genome edited crops in the research and development pipeline (Bohle et al., 2023). Also, as argued by BfN (2021), potential benefits of NGT-based plant varieties with regard to sustainability goals should be subject to verification and thus need to be assessed systematically and following appropriate guidelines.

Similarly, as for transgenic organisms, potential environmental risks may be associated with the introduction of genome-edited organisms. The spread of NGT organisms and/or spread of the modified genes into wild populations and communities may have unpredictable and possibly adverse consequences for the exposed ecosystems (Snow et al., 2005; Ellstrand et al., 2013). NGT products may cause potential adverse effects as a result of intended genetic modifications and unintended genomic alterations introduced during their development, in particular ‘off-target’ and unintended ‘on-target’ mutations (see, e.g., Kawall et al., 2020; Chu and Agapito-Tenfen, 2022; AK, 2023). While such unintended genomic modifications may be eliminated in plant breeding via repeated backcrossing during variety development, they are considerably less easily removed in animal breeding when introduced in founder animals. Thus, the risk assessment conducted for NGT animals and in particular their phenotypic assessment needs to thoroughly address whether any adverse effects result from such unintended modifications.

Overall, the increased use of NGTs is expected to lead to a wider spectrum of GMOs on the market (Broothaerts et al., 2021; Parisi and Rodríguez-Cerezo, 2021; VKM, 2021). Significant challenges regarding the assessment of potential negative effects on the environment and human and animal health have been identified for GM plants (Naegeli et al., 2021) and GM microorganisms (GMMs) (More et al., 2020) produced with NGTs. While evidence for potential applications of NGTs in crop plants has been gathered (see, e.g., Eckerstorfer et al., 2019; Menz et al., 2020; Unkel et al., 2020; Gelinsky, 2022; Bohle et al., 2023), respective work on overviews for other organisms has just started (EC, 2018; 2022).

The diversity of potential applications and the numeric increase in developments poses challenges for competent authorities and risk assessment bodies at EU and national levels. The European Commission (EC) has considered knowledge on plants produced with NGT sufficient to develop a regulatory proposal for plants obtained by certain NGTs (EC, 2021), published in July 2023 (EC, 2023b; 2023c). However, both the study and the proposal have been contested (BfN, 2021; Spranger, 2023b). In the meanwhile, the EC has mandated EFSA to produce scientific opinions on GMMs (EC, 2022; Kagkli, 2023) and on new developments in biotechnology applied to animals (including synthetic biology and new genomic techniques) (EC, 2018; Ardizzone, 2023), to identify potential novel hazards compared to established techniques of genetic modification, and to determine the adequacy of existing guidelines for risk assessment or need for updated guidance.

An improved overview on current developments concerning GMOs is thus necessary for the further development of the existing guidance for risk assessment, in particular for the assessment of potential environmental effects. The initiative to implement a mechanism for a broad and regular horizon scanning to monitor and assess developments of synthetic biology under the framework of the Convention on Biological Diversity underlines this need (CBD, 2018). Conversely, HS as carried out in this study provides relevant input to ongoing Convention of Biological Diversity HS and assessment processes on synthetic biology (CBD, 2023).

Thus, the objective of the HS conducted in the framework of this study was to identify potential future applications in four groups of organisms, GM terrestrial animals, GM fish, GM algae and GMMs, and to provide the necessary overview on the developmental stage and the various fields of potential applications. Insects and applications with gene drives were excluded from this HS. No gene drive GMOs have to date been released into the environment, and there are significant obstacles to such releases on the technical as well as societal and regulatory levels. Research on gene drive GMOs and challenges for their risk assessment have been described, e.g., by the National Academies of Sciences, Engineering, and Medicine (NAS, 2016), by scientific associations in Europe (CSS, 2019) and (for insects only) by the EFSA GMO Panel (EFSA, 2020). Significant research and development also pertains to GM insects without gene drives (Evans et al., 2019; Pare Toe et al., 2021; Waltz, 2021), which warrant separate HS studies. Viruses, which are also sometimes considered microorganisms, were excluded from this HS, because we recently conducted a separate HS on viruses (Eckerstorfer et al., 2014).

We focused on applications intended for release into the environment and included GMOs irrespectively of the methods used to produce them, particularly including GMOs produced by NGTs like genome editing. We have aimed at identifying ongoing developments and potential future applications on an individual level, i.e., a specific trait achieved by genetic modification in a certain species for a specific purpose. An analysis of the scientific literature published within the last 10 years (until the end of January 2023) constitutes the basis of our work. In addition, we have considered information retrieved from grey literature, official websites, and databases.

## 2 Materials and methods

**FIGURE 1:**
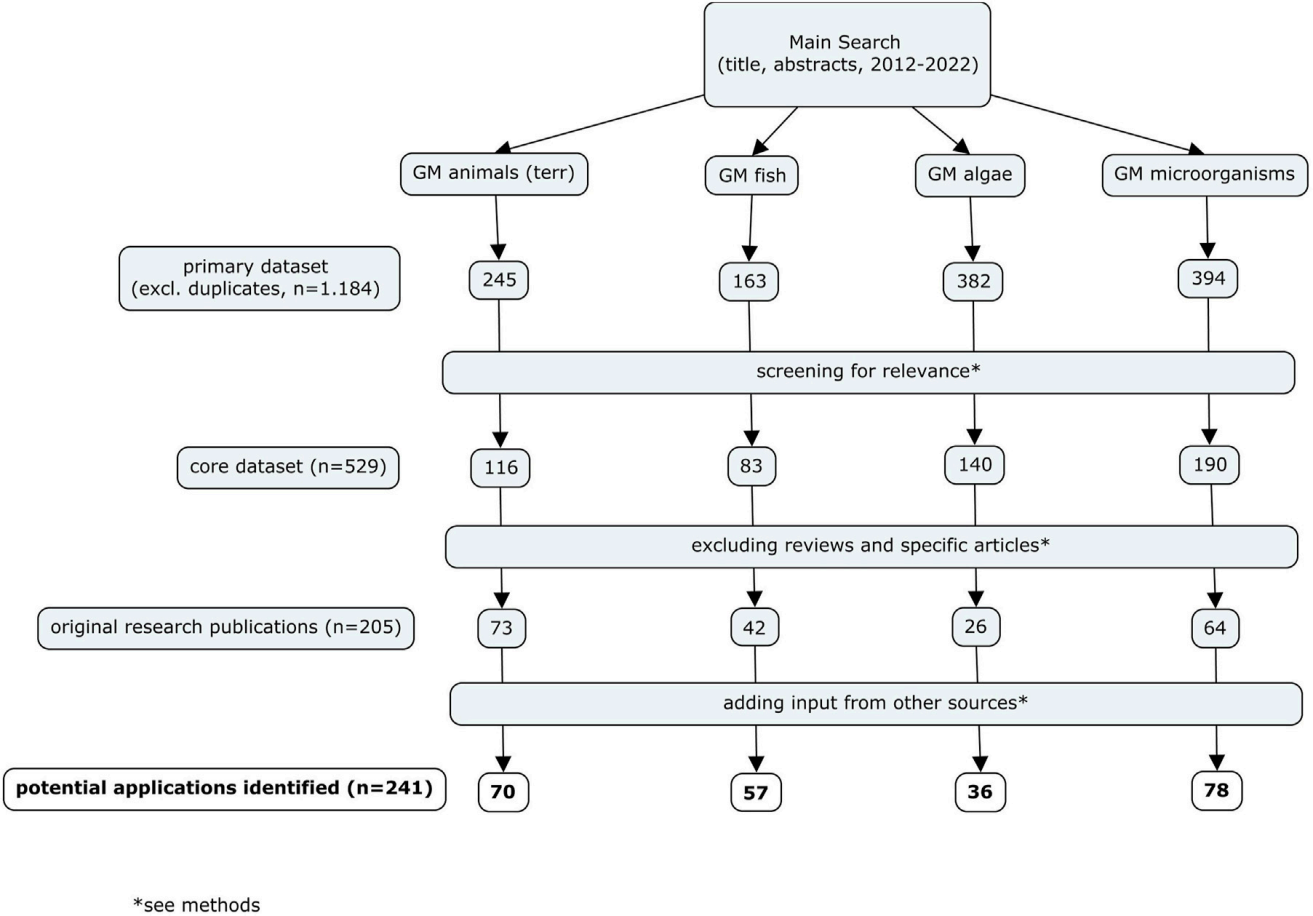
Flowchart of the search strategy.

A HS was conducted to identify potential future GM applications with environmental relevance developed for terrestrial animals (excluding insects and gene drives), fish, microalgae and microorganisms. We addressed this task by focusing on two pillars: A survey of peer-reviewed scientific articles and an additional search for relevant information covering grey literature (e.g., reports), publicly available information of regulatory authorities on market applications and field trials as well as requests for information addressed to responsible experts from selected national competent authorities in non-EU countries. The identified applications were screened for relevance and evaluated with respect to their development status according to defined criteria. Figure 1 provides an overview of the search strategy and the approach for the analyses of the results.

### 2.1 Search strategy

The literature search was conducted at the end of January and in early February 2023 by use of Scopus, an Elsevier’s abstract and citation database. Separate searches were conducted for each group of GMOs. All hits were imported into Citavi 6, a software tool for reference management and knowledge organization, and duplicates were removed upon import. Selected keywords were arranged in three to five categories varying between groups of organisms: ‘intervention’, ‘organism’, ‘trait’, ‘application’ and ‘general’ (Supplementary Tables S1–S4). All keywords and synonyms within a certain keyword category were combined with the Boolean operator ‘OR’. Each search string had to contain keywords from at least three keyword categories combined with the Boolean operator ‘AND’ in order to limit the number of hits to a manageable number. In addition, the application of filters provided by the search mask were used in all cases to limit the search to research (including conference papers) as well as review articles, articles published between 2012-2022 as well as to English language articles. With respect to the subject area (e.g., ‘agricultural and biological sciences’, ‘environmental sciences’), however, filters were set slightly differently depending on the organism group in order to focus on environmental applications (see also 2.2).

Review articles were used to complement the scientific literature search by screening the most recent, i.e., published after 2019, reviews with focus on the relevant GM applications in order to identify additional original literature (Deng et al., 2014; Gao et al., 2019; Blix et al., 2021; Sproles et al., 2021; Tran et al., 2021; Liu et al., 2022; Sproles et al., 2022; Wani et al., 2023). If review articles contained tables summarizing GM applications, we abstained from adding these to our tables, as they are already published in a review format and did not reveal any new fields of application. This was in particular the case for applications of GMMs for bioremediation purposes (Pant et al., 2021; Tran et al., 2021; Sharma et al., 2022; Rafeeq et al., 2023).

In a second step, grey literature from the following information sources were screened: Reports submitted to or published by the EC (Cows et al., 2010; van der Vlugt, 2020; Broothaerts et al., 2021; Parisi and Rodríguez-Cerezo, 2021), reports published by national scientific academies (National Academies of Sciences, 2017), national reports of OECD member states to the OECD working party on harmonization of regulatory oversight in biotechnology (WP-HROB) and annual WP-HROB updates on relevant developments in member or observer countries (OECD, 2021; 2022; 2023) as well as information available online, as, for example, the website of the Genetic Literacy Project (GLP) and its global gene editing regulation tracker (Genetic Literacy Project, 2022).

The search results were also complemented with information on market applications and field trials from regulatory databases and websites of national competent authorities of selected non-EU countries. We focused on information from countries which are known to encourage the development of biotechnology products and provide public information concerning such products (e.g., Australia, Canada, Brazil, US). As publicly available information varies among countries (with respect to type and detail of information as well as regarding accessibility and language) and due the complexity of the different regulatory systems, we chose to complement and verify the online search with requests for information addressed to official national experts from the OCED WP-HROB network (e.g., experts from the US, Canada, Brazil, Argentina, Japan and Australia). In addition, we screened the GMO register of the EC on part B notifications on GMOs other than plants, i.e., experimental releases (EC, 2023a).

### 2.2 Screening for relevance

**FIGURE 2:**
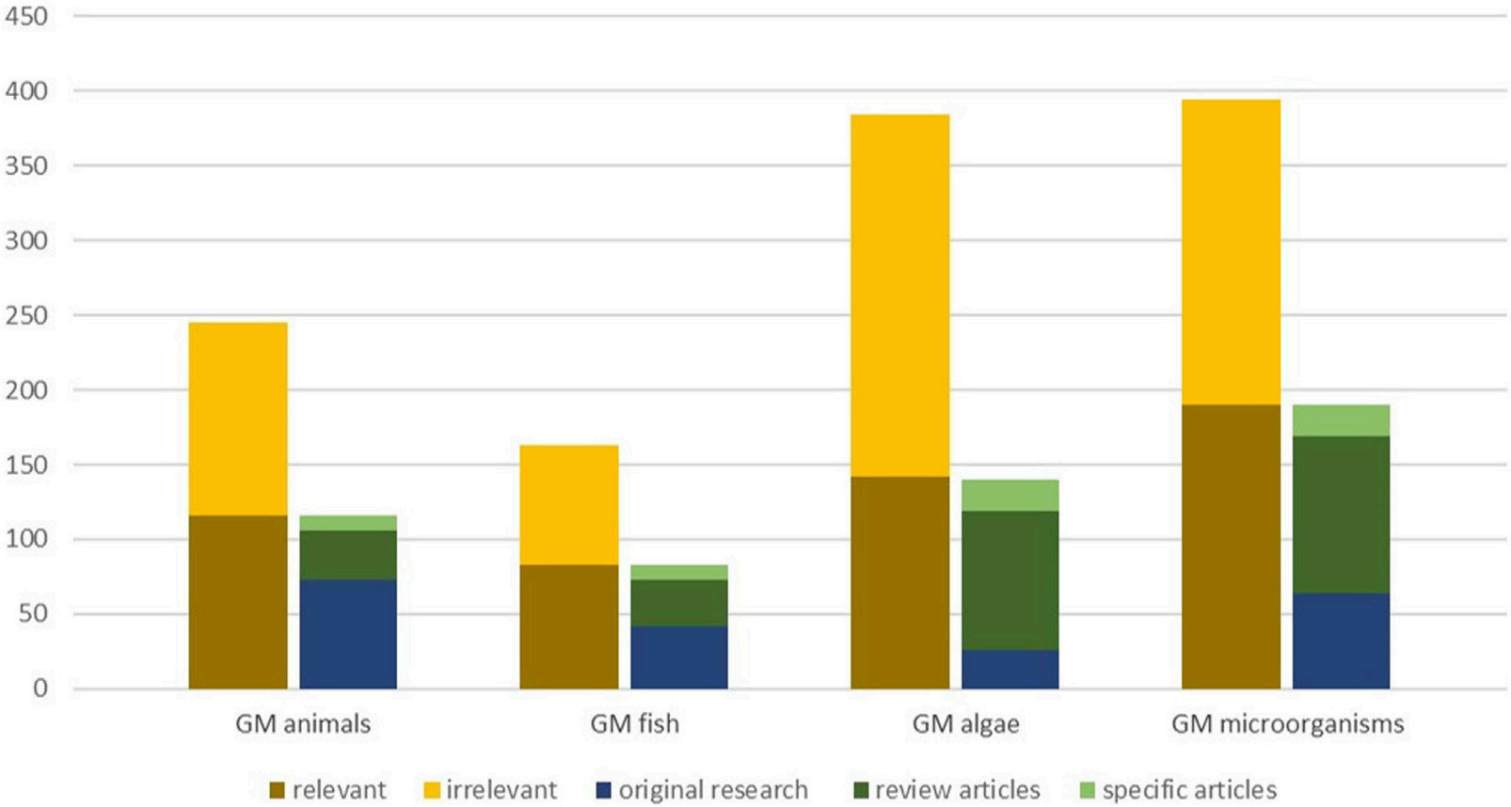
Overview on screening of articles for relevance (first column displays number of relevant and irrelevant articles identified); Categorisation of relevant articles (second column displays original research, review articles and specific articles with a focus on risk assessment/biosafety issues, sustainability, technology assessment or regulatory aspects).

For each group of organisms, two experts involved in the HS independently screened the titles and abstracts of the retrieved articles according to the chosen relevance criteria. The same criteria were applied for the screening of review articles and grey literature. The screening was conducted by experts chosen with regard to their expertise, which allowed exploitation of the specific expertise of all experts involved, to minimize expert bias and avoid inadvertent mistakes. Relevant articles were further classified into two categories: original research and review articles (Figure 2). In addition, articles dealing with issues of risk assessment, biosafety, sustainability, regulation, or technology assessment were marked as specific articles. These mostly include review articles, but also original research investigating respective issues. In some cases, uncertainties regarding relevance could be clarified by analysis of the full text and/or were discussed among experts until inter-reviewer agreement was reached according to a consistent use of the relevance criteria listed as follows:

Relevant

• Studies in which either established techniques of genetic modification or NGTs were used to alter the genome of the respective organism.
• Studies on respective GMOs with traits of market-relevance, such as increased growth or disease resistance.
• Studies on respective GMOs targeting the use in the environment, which are subject to Directive 2001/18/EC in the EU.
• Studies on respective GMOs available on the market, or authorized for marketing or field testing (e.g., Atlantic salmon or *Tilapia* sp. with enhanced growth characteristics, hornless or heat tolerant cattle).

Not relevant

• Studies on GMMs for contained use according to Directive 2009/41/EC (e.g., in bioreactors). This includes studies aiming at the metabolic engineering of GMMs and GM algae with the aim of producing biological substances (e.g., enzymes, fatty acids, antigens) in contained facilities, e.g., in the food, feed or pharmaceutical industry.
• Studies in which DNA sequencing was conducted to elucidate the genomic diversity of the respective organism group (e.g., detection of genomic variation with next-generation sequencing (NGS) technologies).
• General studies on method development, i.e., the development of transformation or transfection methods for certain species or taxonomic groups.
• General gene function studies, i.e., studies or screens with the purpose to elucidate the function of genes or the functioning of certain metabolic pathways (e.g., heavy metal detoxification, salt tolerance) not specifically targeting a marketable trait. This also includes studies aiming at the elucidation of communication and interaction mechanisms between species (e.g., between plants and microorganisms like pathogens, mycorrhiza or endophytes).
• Studies concerning the development of detection methods, i.e., studies dealing with the identification and detection of GM modifications or biological substances (biosensors like e.g., riboswitches).
• Studies on applications in human medicine, e.g., studies with GM pigs for xenotransplantation purposes. With respect to veterinary medicine we excluded the application of GM vaccines as these were included in previous work (Eckerstorfer et al., 2014). Studies dealing with disease resistance traits towards animal disease agents were, however, considered relevant. Other applications in the field of medicine would most probably be subject to strict hygiene requirements and thus held in closed facilities excluding contact with the environment.
• Studies on GM insects and gene drive applications (e.g., GM mosquitos), which were excluded from the scope of our HS.

However, sometimes it was challenging to evaluate the environmental relevance of potential applications. Modifications of GM algae mainly concern composition, in particular the enhancement of cellular lipid yield. Such traits are relevant for applications in food industry, e.g., the production of polyunsaturated fatty acids (PUFAs) as food supplements, as well as for the production of biofuels. Compared to applications in the food industry, large scale production of biofuels may involve the cultivation of GM microalgae in open pond systems accompanied by environmental exposure. Therefore, only applications of GM algae which were developed for biofuel production as a main purpose were taken into account in this HS.

### 2.3 Data analysis and evidence synthesis

The identified articles were independently assessed by two experts for relevant applications of GMOs, their status of development and classified according to different fields of application (e.g., bioremediation, biocontrol, disease control, reproduction). In this HS a particular application was defined as a targeted trait within one species. If the same trait was targeted in two different species, it was counted as two applications. The application of the same trait in the same species, but at different stages of development, was counted only once referring to the latest stage of development. However, sometimes different mechanisms or modifications within the organisms were targeted, in order to achieve a certain trait. In these cases, the respective trait/species combinations were counted as separate applications. For example, the trait heat tolerance in cattle refers to two different applications: Laible et al., 2020 edited Holstein Friesian cattle for diluted coat color for a higher heat tolerance. Another approach for more heat tolerant cattle is to introduce a single base deletion in the prolactin receptor (PRLR) gene of, e.g., the Senepol breed into other breeds like Holstein Friesian. Animals bearing this mutation have a short and sleek hair coat (SLICK trait) which leads to higher heat tolerance (Porto-Neto et al., 2018). Also, for other traits, different mechanisms have been used in order to reach the desired phenotype in the same species (e.g., enhanced growth, resistance to the porcine reproductive and respiratory syndrome (PRRS), sex reversal, sterility). For GM algae and GMMs a differentiation of the various mechanisms involved to achieve a certain trait was not always possible. This was encountered, e.g., for biofuel applications aiming at the modification of the fatty acid metabolism. These applications were counted separately, probably slightly overestimating the number of applications. In addition, market-relevant applications identified in additional sources were only counted if the respective species and the genetically modified trait could be identified. In case of unspecific information on species, modification or trait, the respective information was not taken into account, thus leading to a slight underestimation of the number of applications of market relevance.

For the assignment of the developmental status to the (potential) GM application identified in the literature search, we chose to apply three categories and distinguished research papers dealing with I) basic research, II) advanced or application-oriented research and III) (near) market development (see below). The decisive criterion for the differentiation between category I and II was the demonstration of the expression of the targeted trait in the respective organism. However, in particular, for unicellular organisms, it was difficult to clearly identify proof of concept studies intended to be assigned to category I. Species like *Chlamydomonas reinhardtii*, *E. coli* and *Sacchcaromyces cervesiae* are used as model organisms in basic research as well as in market applications. However, these ambiguities in our opinion do not significantly impact the results of the overview on potential GM applications presented in this study. A common understanding to ensure consistency was established between all involved experts, which was later refined after the first and before the second round of review.

#### I Basic research

This category covers studies conducted in model expression systems (e.g., yeast, *Escherichia coli*), model species (e.g., the zebrafish, *Danio rerio*, or *Sacharomyces cervesiae*), or in cell or tissue cultures. It comprises studies investigating specific gene functions or gene regulation mechanisms in relevant organisms which may be further developed as market-oriented traits. Studies in this category have more of a scoping character, aiming at exploring the potential for new developments. This includes proof of concept studies to identify candidate genes which may be modified for the development of market-relevant traits at a later stage. For example, some studies with GM algae or GMMs investigated the involvement of certain genes in a certain phenotypic characteristic or metabolic pathways (e.g., lipogenesis and carbon metabolism in microalgae). We assigned studies which do not fulfil all three criteria indicted for category II to this category.

#### II Advanced or application-oriented research

This category includes studies which demonstrate the genetic modification and the function(s) of genes with the purpose of development of a market-relevant trait. However, for GM animals to be assigned to this category, the developed traits had to be demonstrated at the organism level and not only in cells, tissue culture or embryos. Studies of GM algae and GMMs comparing the effect of different genetic modifications on the expression of market-relevant traits were counted in this category. Studies included in this category had to meet all of the following three criteria: a) Use of either established techniques of genetic modification or NGT, b) addressing a specific market-relevant trait, and c) demonstration of the expression of the targeted trait in the respective GMO.

#### III (Near-) Market development

Studies which deal with GMOs that are tested in or released into the environment are included in this category. These could be GMOs already released into the environment, e.g., in field trials or available on the market in EU or non-EU countries. In addition, we assigned original research articles on GMOs to this category, which broadly investigated biological parameters of the GMO beside the intended trait, e.g., growth hormone in GM homozygous and hemizygous Carp (Zhong et al., 2012), or traits of relevance for the risk assessment, e.g., specific traits aimed at the biological containment of the organism like GM algae expressing a phosphite dehydrogenase (Inoue et al., 2022).

## 3 Results

We identified a range of articles, which indicate many different potential future applications of GMOs. Overall, we identified 245 scientific articles on GM terrestrial animals (excluding insects and gene drive GMOs), 163 on GM fish, 382 on GM algae and 394 on GMMs in the Scopus search (Figure 1). Upon screening for relevance, about half of the articles retrieved for each group of GMOs, except for GM algae, were considered as relevant for the purpose of this study. For GM algae, only slightly more than a third of the identified articles were determined as relevant for the objective of the HS (Figure 2).

The HS focused on potential future applications for release into the environment. Therefore, we excluded applications clearly aiming at the contained use of GMOs. This concerned in particular research studies dealing with GM algae and GMMs, the majority of which report on their use as biorefineries for a range of improved or novel bio-substances (e.g., enzymes, fatty acids, antigens, pigments) in the food, feed or pharmaceutical industry. For example, GM *Saccharomyces cervesiae* is used in the food industry for bioethanol and biofuel production (OECD, 2023). These applications were disregarded as well as GM algae applications aiming at the production of PUFAs (Songe et al., 2021), phytase (Erpel et al., 2019), carotenoids (Ren et al., 2021), terpenoids (Zhao et al., 2020), astaxanthin (Zheng et al., 2017), xylitol (Pourmir et al., 2013) and squalene (Kajikawa et al. , 2015). Due to uncertainties regarding the exclusive use of GM algae with modified lipid metabolism in contained systems, the number of articles of GM algae aiming at biofuel production from our analysis represents a conservative assessment (see 2 and Figure 4C). For GM terrestrial animals, important fields of applications, which are beyond the scope of this HS, are their use as ‘bioreactors’, i.e., for the production of biopharmaceuticals (Rehbinder et al., 2009) or as organ donors.

During the screening we classified relevant articles into two types: original research articles (including conference papers) and review articles. Articles of both kinds dealing with risk assessment, biosafety or sustainability aspects were marked (Figure 1). The majority of research articles was found for GM terrestrial animals (73) and GMMs (64), the least for GM algae (26). We also retrieved a significant number of review articles, in particular for GM algae and GMMs. However, most of the reviews were not useful for our purposes, because they do not report on GM applications at trait and organism level or do not allow the assignment of a field of application to a GMO. In fact, they address a wide range of different aspects: specific organism (e.g., exiguobacteria), specific degradation mechanisms for certain groups of compounds (e.g., polyaromatic hydrocarbons), specific issues of environmental contamination (e.g., arsenic contamination of ground water), or biological control (e.g., control of cockroaches). Regarding the engineering of microalgae for biofuel production various genes of interest (Rock et al., 2021), strategies for modifications of pathways for improving lipogenesis in microalgae (Fathy et al., 2022) or of methodological aspects of genetic manipulation (Shokravi et al., 2021) were reviewed. However, the level of depth in these reviews did not match our purpose and thus were of limited relevance for the identification of potential applications. A substantial number of reviews mentioned or discussed applications of GMOs (e.g., sex control in animals, bioremediation) as a potential future option (Tarfeen et al., 2022). Some articles focused on ethical aspects of GM terrestrial animals or aspects of societal acceptance of the technology not always referring to specific developments or applications (Ruan et al., 2017; Bruce and Bruce, 2019).

Some of the retrieved publications, in particular those on GM algae and GMMs, dealt with issues related to risk assessment, biosafety and sustainability rather than with the development of specific GMOs. Some addressed risk assessment approaches for GMMs in general (Glandorf 2019) or for certain groups of GMMs (Henley et al., 2013), while other discussed the monitoring and regulation of such applications (Chimata und Bharti 2019; Ryder 2017; Wozniak et al., 2012) or focused on biocontainment (Schmidt und Lorenzo 2012; Mandell et al., 2015; Rovner et al., 2015; Stirling und Silver 2020; Torres et al., 2016; Wright et al., 2013; Motomura et al., 2018). These studies were only taken into account in the analysis if specific applications of GMOs were reported (Motomura et al., 2018; Ishikawa et al., 2021).

**FIGURE 3:**
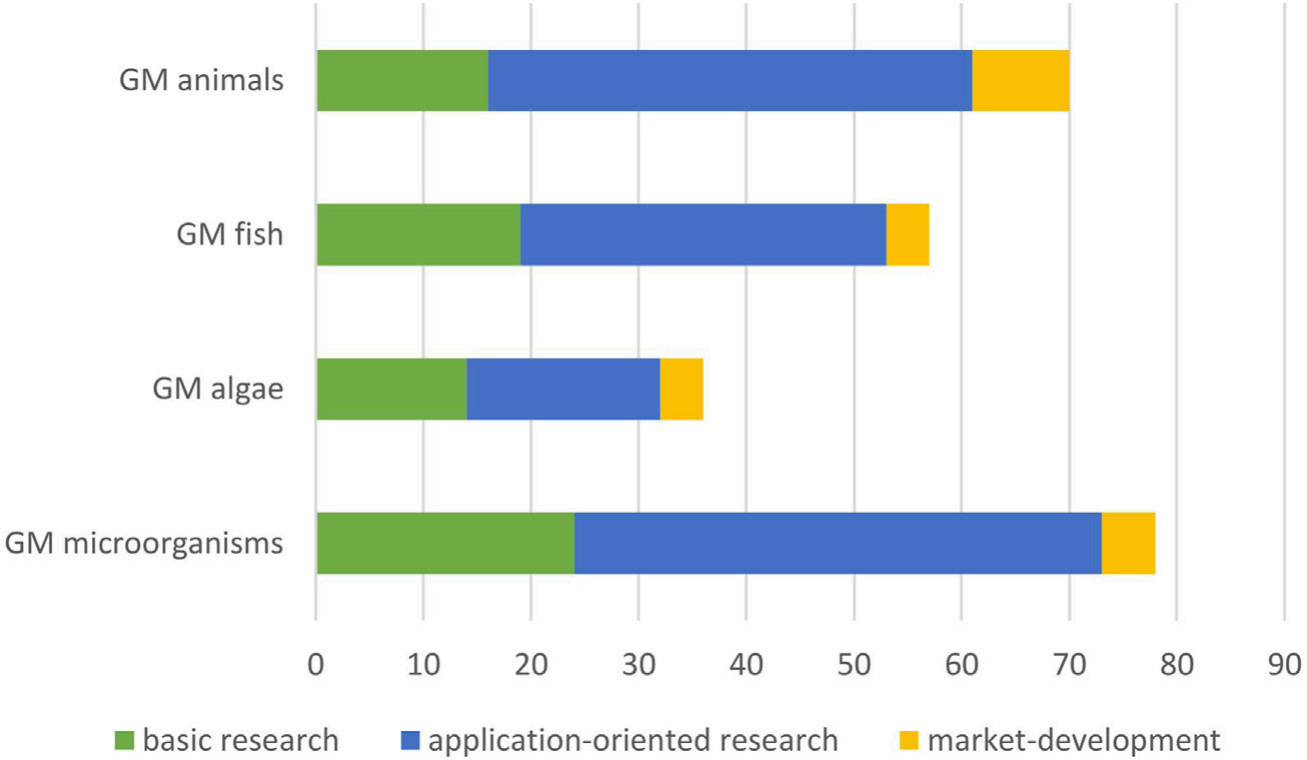
Number of identified applications according to level of development.

From the analysis of identified research articles and the additional information sources we identified overall 70 applications of GM terrestrial animals, 57 of GM fish, 36 of GM algae and 87 of GMMs that are relevant for this HS (Figures 1, 3). These applications were further analysed regarding their field of application and status of development and compiled in tables (Supplementary Tables S5–S25).

### 3.1 Fields of applications

**FIGURE 4:**
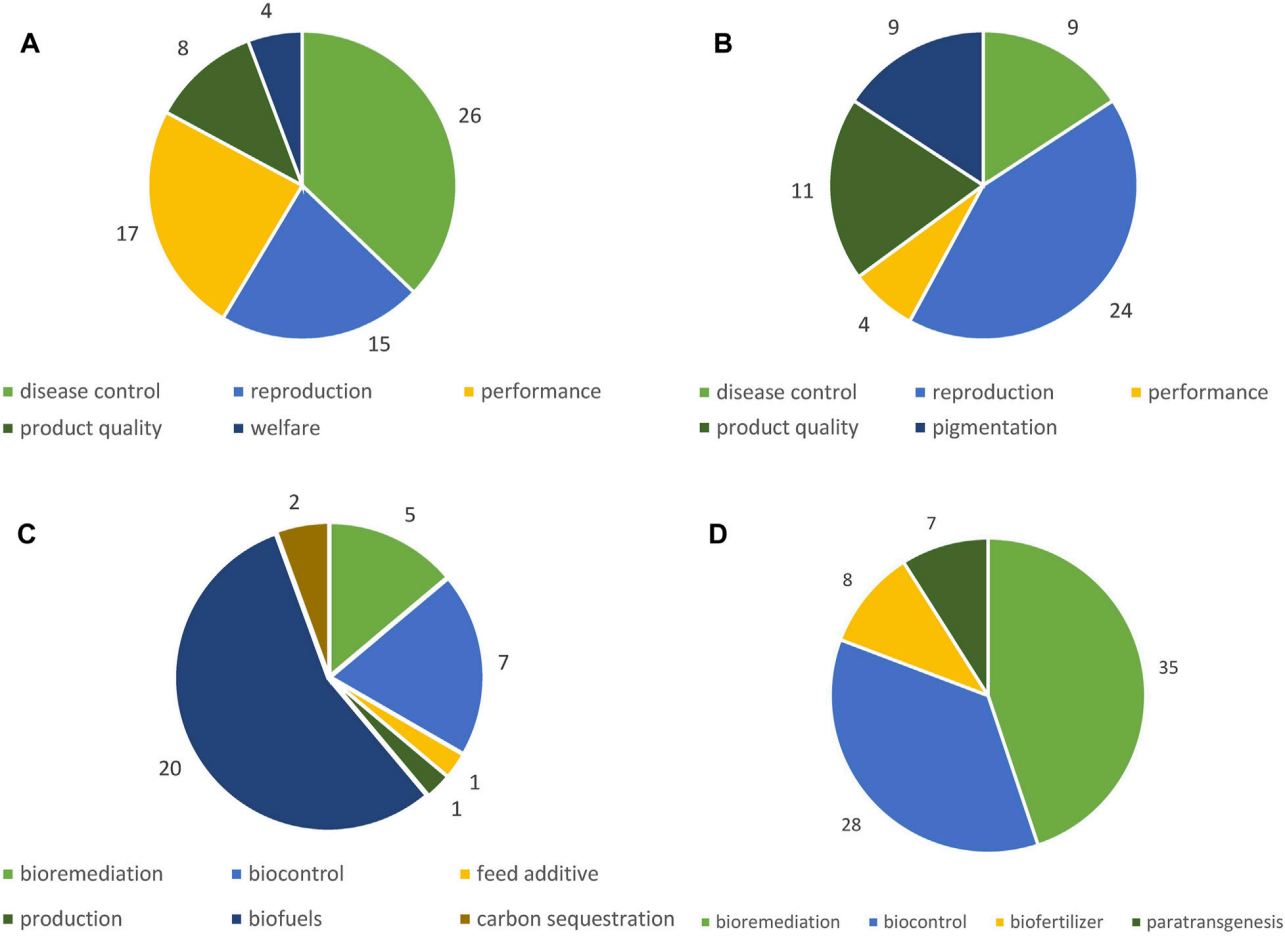
Number of potential applications identified according to field of application. **(A)** GM terrestrial animals, **(B)** GM fish, **(C)** GM algae, **(D)** GM microorganisms.

We classified the identified potential applications of GMOs according to different fields of applications, naturally varying depending on the organism group (Figure 4). Detailed tables listing all identified applications can be found in the Supplementary Material (Supplementary Tables S5–S25).

#### 3.1.1 GM terrestrial animals

**FIGURE 5:**
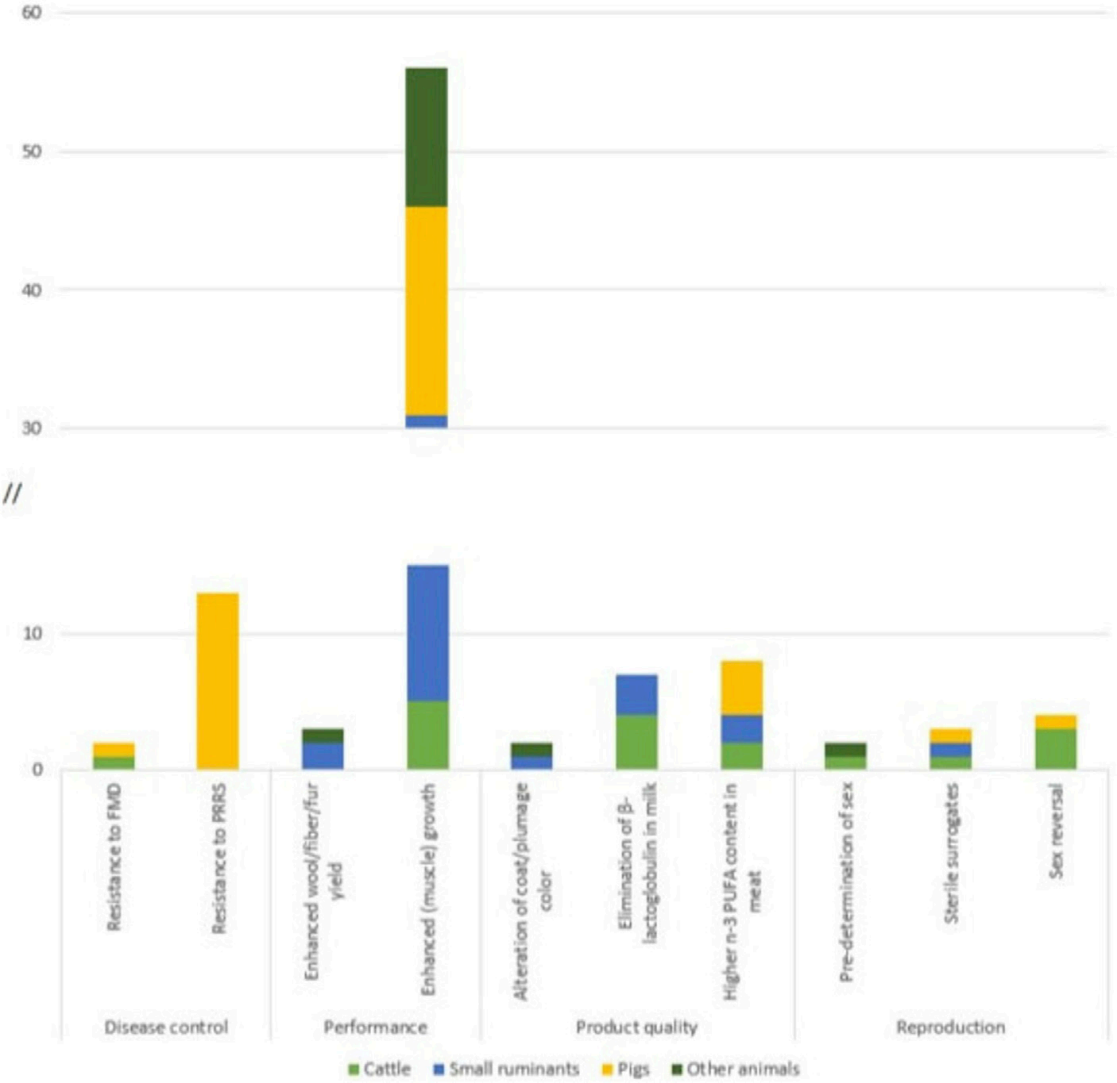
Number of articles identified for GM terrestrial animals according to traits which have been targeted in more than one animal category + the trait resistance to PRRS.

Genetic modifications are particularly performed in farm animals, including cattle, goat, sheep, pig, horse, rabbit, chicken, and quail (Supplementary Tables S5–S13). GM terrestrial animals are generated for various agricultural purposes. Overall, five fields of application were identified for GM terrestrial animals: disease control, performance, reproduction, product quality, and animal welfare (Figure 4A). The by far most often targeted and most broadly applied trait within the field of performance, reported for all livestock species and all stages of development, is enhanced muscle growth (Figure 5). Other applications are pigs with an increase in the amount of lean meat (Zheng et al., 2017; Zeng et al., 2018), increased milk yield in goats (Zhang et al., 2014), increased fiber yield in Cashmere goats (Wang et al., 2016), increased wool yield in sheep (Li et al., 2017) extended hair length in rabbits (Y. Xu et al., 2020) and enhanced weight gain of piglets due to enrichment of sow milk with human α-lactalbumin (Ma et al., 2016).

The trait most often targeted within the field of disease control is the generation of disease resistant or disease resilient animals: resistance to tuberculosis and mastitis in cattle (Liu et al., 2014; Gao et al., 2017), resistance to African swine fever in pigs, or resistance to Avian leukosis virus in chicken (Koslová et al., 2020). The trait targeted the most in a single species is resistance to the porcine reproductive and respiratory syndrome (PRRS) in pigs (McCleary et al., 2020; Y; Xu et al., 2020) (Figure 5).

Targeted traits within the field of animal welfare are: the generation of GM hornless cattle, in order to avoid both painful dehorning and injuries in densely stocked barn environments (Carlson et al., 2016), thermoregulation in cattle (Hansen, 2020) or cold tolerance in pigs (Zheng et al., 2017), and the delay of adolescence in pigs as an alternative to castration (Flórez et al., 2022). However, it is controversial whether these applications can actually increase welfare (Eriksson et al., 2018; De Graeff et al., 2019).

Livestock species are also modified to increase product quality. In the identified applications the purpose is to enhance the product quality of milk, meat, eggs and wool in various ways: the elimination of β-lactoglobulin in cow and goat milk to achieve lower allergenicity of milk (Zhou et al., 2017; Wei et al., 2018), cow milk with low lactose (Su et al., 2018), a higher n-3 PUFAs content (Wu et al., 2012) or goat milk enriched with human-lactoferrin (Cui et al., 2015). In order to improve the nutritional value of meat, the content of n-3 PUFAs is targeted in cattle, sheep and pigs (Cheng et al., 2015; Tang et al., 2019; Luo et al., 2020), intramuscular fat is increased (Gu et al., 2021) and an alpha-gal (Galactose-alpha-1,3-glacatose) free GM pig was developed (Hauschild et al., 2011). Furthermore, the coat color of sheep (Zhang et al., 2017) and the composition of the egg white in chicken (Lee et al., 2020) are modified.

Within the field of reproduction, the two most important traits are sex reversal and sterility. Depending on the species and breed, a certain sex of the offspring is often preferred and pre-determination of the preferred sex may be an opportunity to prevent the culling of animals. Advances are reported in generating all-female offspring in cattle which is the preferred sex in dairy cattle (Xi et al., 2019) and the generation of all-male offspring in beef cattle, in order to produce more meat (Owen et al., 2021). In order to prevent the castration of pigs, GM male pigs with a female phenotype are generated using CRISPR (Kurtz et al., 2021). The purpose of the second trait, sterility, is to generate surrogates for transplantation of allogenic germ cells into either ovaries or testes. The idea is to transplant germ cells of animals with more desirable genetics, in order to enhance production efficiency. This trait is targeted in cattle, goats and pigs (Ideta et al., 2016; Park et al., 2017; Ciccarelli et al., 2020). Furthermore, advances are made for pre-determination of the sex in buffalos (Zhao et al., 2020) and in chicken, in order to identify the sex pre-hatch (Doran et al., 2018).

Overlaps exist regarding the various fields of applications, as some applications may serve two purposes. For example, we classified traits such as sex reversal or sex determination to the category reproduction. However, they might as well be assigned to the category animal welfare, because the determination of the sex of farm animals implies less culling or no castration in case of pigs, if the offspring is all female. Similarly, assignment to the category performance is conceivable, as male cattle perform more efficiently. The trait sterility was assigned to reproduction, but might just as well be an animal welfare trait, as it aims at the reduction of castration in pigs.

#### 3.1.2 GM fish

**FIGURE 6:**
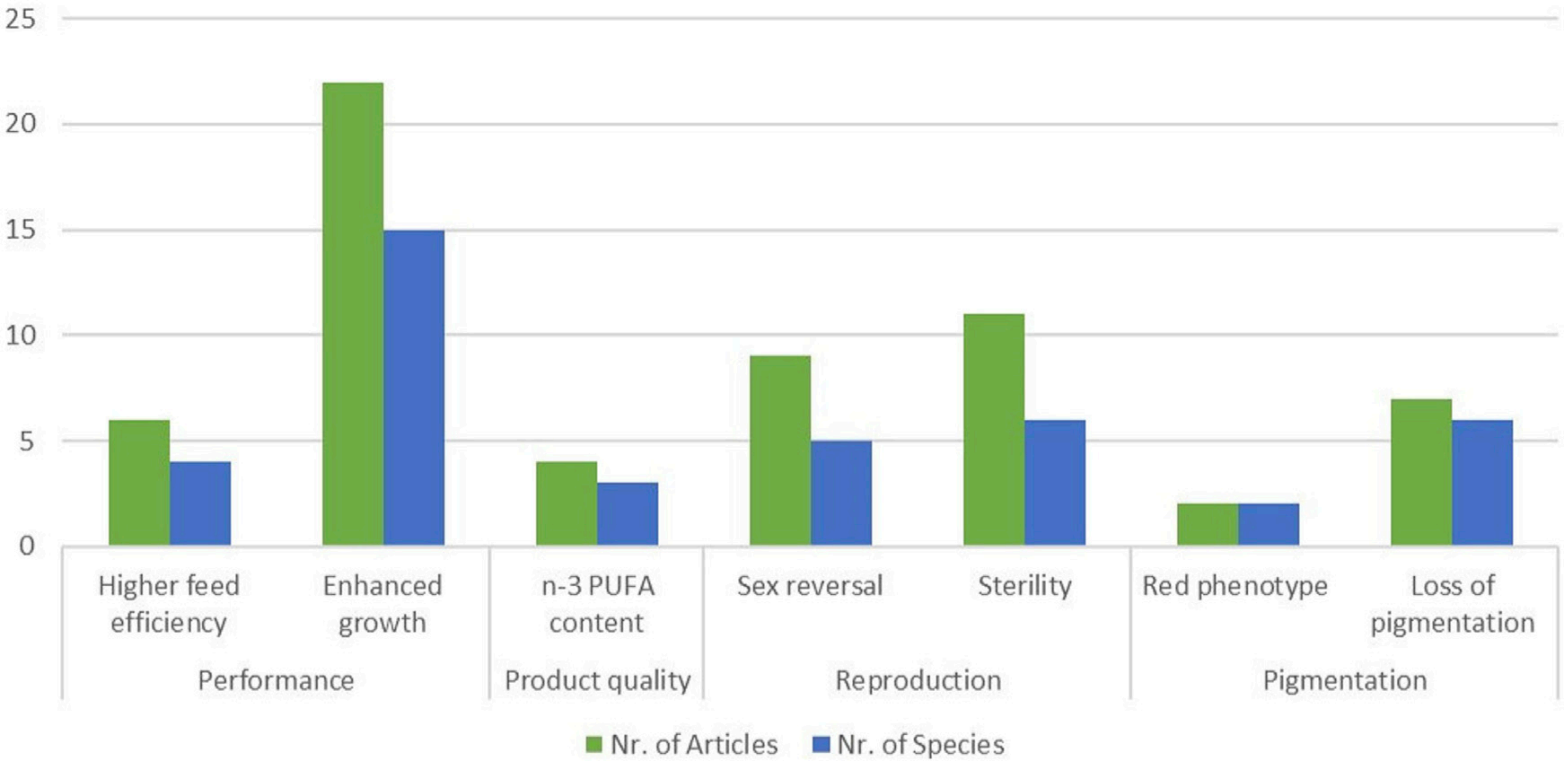
Number of articles identified for GM fish (green columns) and number of species of GM fish according to traits which have been targeted in more than one species.

There is research on a wide range of finfish species including Atlantic salmon, common carp, channel catfish and Nile tilapia (Supplementary Tables S14–S19). The focus is on GM fish with enhanced performance traits for aquacultural purposes, enhanced muscle growth being the dominant trait both with respect to numbers of identified studies as well as with respect to the numbers of species modified (Figure 6).

Five different fields of application were identified for GM fish (Figure 4B): reproduction, product quality, disease control, pigmentation, and performance. The main trait targeted within the field of disease control is the generation of disease resistant or disease resilient fish, which was reported for channel catfish (Abass et al., 2022; Coogan et al., 2022), grass carp (Ma et al., 2018), Nile tilapia (Chiang et al., 2020), rainbow trout (Chiou et al., 2014; Lo et al., 2014), zebrafish (Wang et al., 2014; Chengfei et al., 2017), Asian sea bass (Yang et al., 2021), Rohu carp (Chakrapani et al., 2016), and chinook salmon (Dehler et al., 2019).

The most targeted trait within the field of performance is enhanced muscle growth, which is reported for various fish species: Atlantic salmon (Tibbetts et al., 2013), blunt snout bream (Jiang et al., 2017; Sun et al., 2020), channel catfish (Khalil et al., 2017; Coogan et al., 2022), common carp (Zhong et al., 2016), Nile tilapia (Wu et al., 2023) and other species (Supplementary Tables S14–S19). A trait which is related to enhanced growth, is improved feed efficiency, which is specifically reported for Atlantic salmon (Tibbetts et al., 2013), gibel carp (Huang et al., 2021), Nile tilapia (Wu et al., 2023), and red sea bream (Ohama et al., 2020; Washio et al., 2021). A behavioural trait targeted in Pacific bluefin tuna could be identified: GM tuna showed slower swimming behaviour, which might reduce losses of GM tuna from sea cages to the open sea (Higuchi et al., 2019). In order to improve product quality, especially meat quality, the content of n-3 PUFAs is increased in channel catfish (Xing et al., 2022; Xing et al., 2023). The same trait is altered in terrestrial farm animals (see previous section) and in the model fish *D. rerio* (Pang et al., 2014). In the zebrafish model is also the loss of undesirable intermuscular bones is targeted (Nie et al., 2021).

Within the field of reproduction, we identified two main targeted traits: sterility and sex reversal. Sterility in fish is meant to prevent genetic introgression of farmed animals into wild populations (Wargelius, 2019). It is reported for Atlantic salmon (Wargelius et al., 2016; Kleppe et al., 2022), common carp (Su et al., 2014), channel catfish (Qin et al., 2016), Nile tilapia (Tao et al., 2020), sterlet (Baloch et al., 2019) and the model zebrafish (Zhou et al., 2018). Sex reversal is especially of interest to generate the sex which leads to the higher meat yield. Depending on the fish species this can either be the female, e.g., common carp (Zhai et al., 2022) or the male fish, e.g., Nile tilapia (Stokstad, 2020) or yellow catfish (Dan et al., 2018). This trait is also reported for the model fish Medaka (Luo et al., 2015) and zebrafish (Zhou et al., 2018).

Pigmentation is an important economic trait in farmed fish, because there are certain phenotypes which have a higher economic value than others (Luo et al., 2021). Applications targeting pigmentation are reported for Nile tilapia (Wang et al., 2022; Wang et al., 2023) and yellow river carp (Jiang et al., 2022). Furthermore, the loss of pigmentation is targeted in Atlantic salmon (Edvardsen et al., 2014), common carp (Mandal et al., 2020), large-scale loach (X. Xu et al., 2019), Medaka (Fang et al., 2018), white crucian carp (Liu et al., 2019) and zebrafish (Irion et al., 2014).

#### 3.1.3 GM algae

All articles identified reported work on unicellular algae, i.e., microalgae. Articles on macroscopic algae, e.g., brown algae, i.e., seeweeds, were not found. GM microalgae include species like *Chlamydomonas* sp. and *Chlorella* sp. as well as a few diatoms, such as *Nannochloropsis* sp. and *Phaeodactylum tricornutum* (Supplementary Table S20). The focus of our study was on GM microalgae with the following traits (Figure 4C): production of lipids or triacylglycerols for use as or in biofuels, increased CO_2_ sequestration, increased efficiency of the photosynthetic capacity and algal biomass productivity, hydrocarbon production, and wastewater treatment (see overviews in, Geraldi et al. (2023); Thanigaivel et al. (2023); Barati et al. (2021); Singh and Ghosh (2021)). At the current stage it is not foreseeable whether these applications will be cultivated in open-culture systems or closed photo-bioreactors. In Europe, both types of cultivation systems are possible (Enzing et al., 2014), consequently open pond cultivation (e.g., raceway ponds) cannot be excluded for GM microalgae applications with the above-mentioned traits in the future. Open and closed culture systems of microalgae have different advantages and disadvantages depending on the taxon, the specific applications and the local conditions (Mata et al., 2010). However, as the information provided in title and abstract of articles sometimes lacks information on the specific purpose of applications (e.g., for studies on algal metabolism), our data may slightly underestimate the number of potential biofuel applications (see 2).

Applications of GM microalgae for use in the environment comprise traits for bioremediation and environmental restoration, in particular of aquatic ecosystems, as well as for control of human pathogen vectors or disease control (bacterial or viral diseases) of aquatic animals (Figure 4C). Specifically, several applications producing recombinant oral vaccines (e.g., antigens, antimicrobials) or double-stranded RNA are being developed. Only one application assessed GM microalgae under field conditions in an open pond (Inoue et al., 2022). The GM microalgae expressed a phosphite dehydrogenase, in order to be able to utilize phosphite (H_3_PO_3_) instead of phosphate (H_3_PO_4_) as a means of biological containment.

#### 3.1.4 GM microorganisms

The HS identified applications for GMMs (excluding viruses) in various species from different groups of organisms (e.g., bacteria, fungi, and cyanobacteria) (Supplementary Tables S21–S25) addressing a broad scope of different aims (Figure 4D): use as biofertilizers, i.e., as plant growth promoting organisms, and for biocontrol or bioremediation purposes. Several paratransgenesis applications were found, which target the modification of GMMs that are associated with or living in symbiosis with animals (mostly insects) with the purpose to modify relevant characteristics of their animal hosts, e.g., their ability to vector pathogens.

Overall, most of the identified applications are developed for purposes of bioremediation, i.e., targeting contaminations of environmental media such as soil and water. Many employ different bacteria, including many *Enterobacteriacae* and *Pseudomonaceae* serving the enhanced removal of different kinds of pollutants. Organic pollutants targeted include pesticides (Gong et al., 2016; Yi et al., 2016; Xu et al., 2022), antibiotics stemming from industrial and agricultural sources (Liu et al., 2020), and persistent organic pollutants like toluene (Ishikawa et al., 2021), nitrobenzene (Deng et al., 2022), and para-nitrophenol (Huo et al., 2022). Anorganic pollutants targeted include mercury (Priyadarshanee et al., 2022), chrome (Zhou et al., 2020), and arsenic (Maleki and Shahpiri, 2022). Also, fungal species and yeasts are developed, e.g., for waste water treatment (Görner et al., 2016), accumulation of different heavy metals, such as cadmium (Zhang et al., 2021), or to aid the volatilization of arsenic from contaminated soils (Verma et al., 2019; Verma et al., 2021). In addition, GM cyanobacteria are developed for the removal of anorganic waste such as phosphate, ammonium, nitrate and nitrite from the wastewater of shrimp aquaculture (Krasaesueb et al., 2023). Some reviews compiling information on bioremediation, provide tables of GMMs but with insufficient information on the specific applications. These applications were not included for further analysis. Thus, our results are a very conservative estimate of GMMs developed for this respective purpose.

Biocontrol applications include a number of developments in bacteria, e.g., *Burkholderia pyrrocinia* (He et al., 2018) and fungi, e.g., *Beauveria bassiana*, *Metarhizium acridum*, Trichoderma harzianum, *Isaria fumosorosea* (Kim et al., 2013; Hu and Wu, 2016; Xia et al., 2018; Tong et al., 2021; Asgari, 2023), which are modified to enhance the efficacy of known biocontrol agents to control different pests or pathogens. Some of these applications express RNAs which trigger a RNAi-based response in the exposed target pests to achieve a biocontrol effect. Others express different types of effector molecules like chitinases (Xia et al., 2018), glycerate-3-kinase (Tong et al., 2021) or bumblebee venom serin proteases (Kim et al., 2013). However, also bacteria and fungi that are not commonly used for biocontrol may be developed into GM biocontrol agents, e.g., *Escherichia coli* strains with modifications to express RNA-molecules which trigger RNAi mechanisms (Xiong et al., 2013; Vatanparast and Kim, 2017; Niño-Sánchez et al., 2021). Other newly developed biocontrol agents are expressing compounds that are toxic for the targeted pest species, e.g., baker’s yeast *Saccharomyces cerevisiae* overexpressing saccharomycin (Branco et al., 2019), or Pichia pastoris *expressing Cecropin A to control* Alternaria (Zhang et al., 2015). One application in the cyanobacterium Synechococcus targets a viral pathogen (White spot syndrome virus, WSSV) affecting shrimp production by expressing a subunit vaccine (Xu et al., 2021).

Some of the studies on biofertilizers can be considered exploratory studies to understand the involved mechanisms (Pai et al., 2012; Jain and Gralnick, 2021), or to increase the efficiency of root symbiosis as a decisive aspect for biofertilizer applications (García-Tomsig et al., 2022). Others target characteristics in bacteria (Das, 2019; Ambrosio and Curatti, 2021) and cyanobacteria (Chaurasia et al., 2017) which increase the ability of the modified microorganisms to be used as plant growth promoting agents or biofertilizers (Supplementary Tables S21, S24).

The identified paratransgenesis applications explore the use of different insect- and nematode-associated bacteria as possible paratransgenic agents (Elston et al., 2021; Lulamba et al., 2021), in order to control the host animal or pathogens vectored by these animals (Mysore et al., 2017; Arora et al., 2018; Wang and Zou, 2019).

### 3.2 Level of development

All applications of GMOs identified in the Scopus search, or retrieved from selected review articles and additional sources, were classified with respect to their application status into three categories as outlined in chapter 2.3 (Figure 3). Across all groups of organisms, about a third of the research articles were considered to be basic research (category I, Figure 3), for GM fish and GM algae a little more and for GM terrestrial animals a little less. Approximately half of the number of identified articles for GM algae were assigned to category II, i.e., early or advanced research developments, and slightly more than a half in the other groups (Figure 3). Very few applications were considered to be near-market development applications, i.e., category III. Slightly more than 10% for GM terrestrial animal and GM algae and a little less than 10% for GM fish and GMMs. Information from grey literature and additional sources could in all cases be assigned to category III. In the following, the results for the four groups of organisms are presented with a particular focus on relevant market applications.

#### 3.2.1 GM terrestrial animals

Most applications identified in regulatory databases and websites of national competent authorities of selected non-EU countries had already been identified by the literature search. However, the additional information sources provided information on the approval of field trials and commercial use of these applications and this enabled a better determination of their developmental stage. Overall, nine market-relevant applications were identified for GM terrestrial animals. In 2016, a trait conferring resistance of cattle to Bovine respiratory disease was patented in the US (Office of Commercialization, 2016). In December 2020, the US Food and Drug Administration (US FDA) approved transgenic pigs, referred to as ‘GalSafe pigs’, for pork consumption. These pigs have originally been generated for potential therapeutic uses, i.e., as a source of medical products, such as the blood-thinning drug heparin, or as source of tissues for xenotransplantation. The modified pigs do not express alpha-gal sugar (Galactose-alpha-1,3-galactose), which is known to cause allergenic reactions in humans with Alpha-Gal-Syndrome or may cause the rejection of transplanted tissues (US FDA, 2020). In March 2022, the US FDA published a low-risk determination for products from genome-edited PRLR-SLICK cattle, i.e., cattle intended to be heat-tolerant (US FDA, 2022). Other applications for which a risk assessment has been conducted previously are, e.g., the transgenic EnviroPig™ with a lower rate of phosphorus excretion assessed by Canada and genome-edited cattle with enhanced muscle growth in Brazil (Supplementary Table S13). Furthermore, there have been some field trials in Argentina with transgenic cattle which express human growth hormone, human lysozym and lactoferrin or antibodies against rotavirus in milk (OECD, 2022). An additional application, which we had overlooked and therefore not included in our analysis, was the development of five genome-edited male sterile pigs for breeding purposes in the US. The US FDA has granted an investigational use authorisation allowing human consumption of these five animals, excluding commercialisation (OECD, 2023). Furthermore, a patent was granted for GM hens modified to produce lethal phenotype in male bird embryos (WIPO, 2023).

#### 3.2.2 GM fish

For GM fish only one study retrieved by the Scopus search could be assigned to category III, i.e., a transgenic carp modified for growth enhancement (Zhong et al., 2012). However, no information was provided regarding future field trials or any plans concerning commercialisation with this species. Additional research gave insights into four previously developed and highly advanced applications, but no additional modified fish species. In 1989, a transgenic Atlantic salmon, AquAdvantage salmon, was developed by AquaBounty, which grows almost twice as fast as non-GM salmon. It is the first GM animal which has been approved for human consumption in the US, Canada and Brazil (Health Canada, 2016; OECD, 2022; US FDA, 2023). In 2018, a genome-edited growth-enhanced tilapia produced by AquaBounty with the CRISPR technique was evaluated and considered eligible for exemption from GMO regulation in Argentina (The Fish Site, 2018; Genetic Literacy Project, 2019). For the same tilapia a risk assessment was conducted in Brazil by National Technical Commission of Biosafety (CTN Bio) in 2019 (CTN Bio, 2023). In 2021, two genome edited fish applications were approved for commercial sale in Japan: red sea bream and tiger puffer, both genetically modified for growth enhancement (OECD, 2022). After our research in spring 2023, an application for field trials with a sterile Atlantic salmon (VIRGIN^®^ salmon) was submitted in Norway (EC, 2023c), but turned down by the Norwegian authorities as the effectiveness of its sterility is contested (VKM, 2023).

#### 3.2.3 GM algae

Field trials and market applications with GM microalgae comprise applications under strict contained use (e.g., targeting the productions of high oleic microalgae oil for use as food supplement) as well as applications for potential cultivation in open ponds (e.g., for biofuel production). Therefore, similarly as with research articles, we focused on applications potentially to be applied in the environment.

Overall, information is available from the US, Australia, and Brazil concerning the species and traits of four transgenic microalgae applications for confined field trials. For example, in the US, an application for experimental release under the Toxic Substances Control Act (TSCA) was approved by the Environmental Protection Agency (EPA) for the environmental release of GM microalgae *Acutodesmus dimorphus*, which had been modified for enhanced fatty acid synthethis (Szyjka et al., 2017). In Australia, an intentional release of the GM microalgae *Nannochloropsis oceanica* with altered fatty acid composition and the inability to use nitrate as nitrogen source was approved by the Australian Office of the Gene Technology Regulator (OGTR, 2020). Releases of GM microalgae in Brazil potentially aiming at biofuel production are also reported by the OECD (OECD, 2021).

In Europe, one microalgae species was tested in small-scale experiments in the context of an EU research project in the United Kingdom (Hamilton et al., 2015). The GM diatom *P. tricornutum* expressed an enzyme to accumulate high-value omega-3-fatty acids. According to the authors, the GM microalgae were cultivated in photo-bioreactors (550 L), smaller bubble column systems (3.5 L) but also in open-pond system (1,250 L). However, we could not find a respective Part B application, i.e., for experimental release (EC, 2023a). The research was part of an EU research consortium under the EC-FP7-KBBE, genetic improvement of microalgae for value-added products (EC, 2013).

#### 3.2.4 GM microorganisms

In this group of organisms again the majority of applications are approved for industrial production of, e.g., ethanol or substances of pharmaceutical interest in contained production facilities (e.g., in different species of bacteria and fungi such as *Saccharomyces* sp. and *Pichia* sp.). These applications are not intended to be released into the environment and are thus not included here. Applications of GMMs were retrieved from risk assessments, notifications or authorisations in the US, Brazil and Canada. However, our analysis remains incomplete as sometimes databases could not be analysed for our purposes due to limited search options (e.g., not at organism level) or lack of information available (e.g., in English) or in sufficient detail (e.g., with respect to the field of application). One application was identified in the Scopus search: a modified yeast, *S. cerevisiae*, which is expressing an envelope protein of the White Spot Syndrome Virus (WSSV) anchored to the yeast surface for use as an oral vaccine agent in shrimp farming (Le Linh et al., 2021).

In Canada, eight fungi and 13 bacteria have been assessed under the New Substances Notification Regulation (NSNR Organisms) which requires an environmental risk assessment (ERA) to be conducted for novel living microorganisms, including GMMs (Canada, 1999; ECCC, 2023). However, this regulation also covers microorganisms, which are not modified using biotechnology and used in agricultural fields as well as modified microorganisms used or produced in contained systems (e g. GM *E. coli*). Only one of these applications falls under the scope of this study, a transgenic *Pseudomonas putida* applied on surface soil in a field trial for herbicide degradation (ECCC, 2016). The other applications of microorganisms notified in Canada under the NSNR concern the use of modified microorganisms in contained systems (GM yeast, GM *Trichoderma longibrachiatum,* or GM *Trichoderma reesei*), for therapeutic uses in humans (e.g., GM *Listeria monocytogenes*), as well as notifications for agricultural field trials with non-GMMs (e.g., *Rhynchosporum secalis* and *Phlebiopsis gigantea*).

In the US submissions for TSCA Experimental Release Applications (TERAs) are required for environmental introduction of GMMs for commercial research and development purposes. In 2020, a TERA for an *Alphaproteobacteria* was submitted (OECD, 2021). In 2021, TERAs for various strains, including those with a bioluminescent marker protein for investigating microbial colonisation of plants and several strains of bacilli to affect nitrogen production (OECD, 2022), were also submitted. Sayler and Ripp report on a controlled field study investigating a transgenic *Pseudomonas fluroescens* strain in contaminated soil for naphthalene degradation (Sayler and Ripp, 2000).

In Brazil 34 GMMs and 17 derivatives and enzymes (e.g., tryptophan, alpha-amylase) for use as feed additive or industrial application (OECD, 2021) are approved (CTN Bio, 2023). Most of all, they comprise yeast strains, *Saccharomyces cervisiae*, e.g., for ethanol production, and microalgae, *Prototheca moriformis*, for triglyceride production. More specifically, 27 genome edited products are commercially approved as ‘Innovative Genetic Improvement Technologies’, three of which are of environmental relevance: a genome-edited *Klebsiella variícola* modified for ammonium fixation, a genome-edited *E. coli* for use as feed supplement in chicken fattening and a genome-edited *Bacillus thuringiensis israelenses* expressing a β-1,4 endoglucanase from *Bacillus subtilis* to be used as soil conditioner (Supplementary Table S25).

No licenses for dealings involving intentional release (DIRs) involving microorganisms were found in Australia (OGTR, 2023). No commercial approvals of GMMs, only viruses, were reported by Argentina to the OECD in 2020 and 2021 (OECD, 2021; 2022; 2023).

## 4 Discussion

The great research interest in NGTs and their potential applications is reflected in a vast number of scientific publications. However, the development of marketable products takes years, in particular for multicellular organisms, which makes scientific reports on the development of GMOs a fairly good forecast for future applications. This HS identified a broad range of potential applications of GM terrestrial animals, GM fish, GM algae and GMMs for use in the environment, of which only a few are tested or used in the environment at the moment. However, the variety of modified organisms and traits as well as the respective range of applications reveals substantial challenges for regulators, risk assessors and scientific experts, which need to be addressed urgently.

### 4.1 Methodological considerations

Only about half of the publications identified during the literature searches, even less for GM algae, met our relevance criteria. Not all of those publications were original research articles describing potential GM applications (Figure 2), only about two-thirds for GM terrestrial animals, half for GM fish, a fifth for GM algae and a third for GMMs. Although not directly comparable, as we did not conduct a full text analysis, these proportions are comparable to literature searches conducted specifically on genome edited plants (Modrzejewski et al., 2019; Menz et al., 2020). In a recent scientific report conducted on NGTs in animals, only about 10% of the articles identified in the literature search was considered relevant (van Eenennaam, 2023). We found the majority of relevant articles to be reviews or specific articles which use the term ‘genetically modified’ or any of its synonyms (Supplementary Table S1) in the abstract or the indexing of articles, even though the respective article did not specifically report on concrete developments of GMOs. In addition, the separation of articles dealing with applications for use in the environment as opposed to those for use in contained systems or in food and feed products was not always unambiguous, especially for unicellular GMOs (GM algae and GMMs). Overall, the screening of results from literature searches constitutes a labor-intensive, but significant step which has to be weighed against the advantage of broadening the basis of literature sources searched (e.g., Web of Science, PubMed, CAB Abstracts).

The JRC study on market applications of NGTs focused on a survey of technology developers, instead of a search in the scientific literature (Parisi and Rodríguez-Cerezo, 2021). However, this approach has the disadvantage that for confidentiality reasons the results could not be presented for single applications, but only in an aggregated form. Information from industry is highly relevant for a HS, as this more clearly indicates the direction of market-relevant efforts undertaken. However, a broad participation of respective companies is not easy to achieve (Parisi and Rodríguez-Cerezo, 2021) and the information gathered on the traits and introduced modifications of the GMOs may not be sufficient to provide risk assessors with appropriate information on emerging GM applications.

### 4.2 Emergence of manifold applications of GMOs in the environment

With respect to GM terrestrial animals, our results show a clear focus on GM farmed animals, i.e., on livestock species predominantly used worldwide for food production, i.e., cattle and pigs, followed by small ruminants, reflecting their worldwide economic importance. Our HS did not identify GM applications in wild terrestrial vertebrates or research and development resulting in respective GMOs within the scope of our study (i.e., excluding gene drives). In general, such applications are still at a conceptual stage or at the very early stages of development, which were not picked up by our search strategy. This also holds for applications pertaining to scenarios brought forward for GM animals to serve species conservation (Redford et al., 2014; Redford et al., 2019; Novak et al., 2018; Kosch et al., 2019; Kosch et al., 2022; Samuel et al., 2020; Macfarlane et al., 2022; see also BfN, 2022).

Developers of GM animals predominantly target traits which are relevant to the production of livestock, in particular disease resistance, performance and reproduction. A Joint Research Centre (JRC) study which amongst others investigated potential market applications of GM animals developed with NGTs, also identified applications for increased biotic stress tolerance and improved meat yield as the focal fields for development (Parisi und Rodríguez-Cerezo 2021). A recent publication by EFSA reports similar findings with an additional focus on reproductive traits (van Eenennaam, 2023). By far the highest number of publications was identified for enhanced muscle growth, with developments in many different species, ranging from ruminants and pigs to chicken, quail, rabbits and even horses (Figure 5). This trait is usually achieved by genetic modifications, i.e., knock out of the myostatin gene. Due to its great economic relevance (see, for example, Nathues et al., 2017) and the enormous challenges of controlling PRRS at a larger scale (see, for example, Rowland and Morrison, 2012; Amadori et al., 2021), resistance to PRRS in pigs is the most frequently targeted trait in a single species (see Figure 5; Chen et al., 2019; McCleary et al., 2020; K; Xu et al., 2020). The apparent focus of work on these GM traits is in line with economic interests driving genetic modifications in livestock.

In GM fish, most GM applications target traits relevant for reproduction, e.g., sex reversal and sterility. The development of sterile fish is considered a prerequisite step for GM applications involving intended environmental release, in order to prevent or minimize gene transfer to related wild populations (VKM, 2021). However, for applications directed to sex reversal, the ultimate purpose of the modification is achieving a higher meat yield. Taking this into account, the yield trait enhanced growth was, as for GM terrestrial animals, the dominant trait in GM fish, modified in various species (Figure 6). In a recent review, Gutási et al., 20023 particularly highlight potential applications in fish medicine (Gutasi et al., 2023). The JRC study, which focused on developments of NGTs in the industrial sector, identified only seven aquatic animals, including six fish species and corals (Parisi and Rodríguez-Cerezo, 2021). Reviews of the scientific literature however revealed far more applications, i.e. 47 of GM fish in this HS and 56 of genome edited fish in the EFSA scientific report on NGT animals (van Eenennaam, 2023).

The majority of developments of GM algae aims at biofuel production. However, substantial challenges of large-scale production (e.g., in open pond systems) regarding environmental safety as well as cost-effectiveness still have to be overcome (Abdullah et al., 2019; Mobin et al., 2022). In addition, applications for other types of uses in the environment, such as the restoration of water sources or disease control in aquatic animals, were found.

For GMMs, similar fields of potential applications as in previous work (van der Vlugt, 2020; Parisi and Rodríguez-Cerezo, 2021; VKM, 2021) were identified. However, we found substantially more applications, in particular with respect to bioremediation (34) and biocontrol purposes (31). Van der Vlugt (2020) identified 11 GMMs and only one commercial application other than for food and feed use, i.e., a soil bacterium for use as fertilizer (van der Vlugt, 2020). Parisi and Rodríguez-Cerezo (2021) found applications of the same soil bacterium and an endophyte fungus, but due to confidentiality reason could not present applications of other soil bacteria and probiotics. However, incompletely disclosed by Parisi and Rodríguez-Cerezo (2021), the limited number of applications of GMMs for use in the environment identified in these two studies compared to the great number identified here (78) is astonishing. As far as the JRC study is concerned, these differences can to some extent be explained by differences in the methodological approach. It focused on the consultation of experts and a survey of public and private technology developers, in order to identify products already being marketed or at a confirmed pre-market development stage (Parisi and Rodríguez-Cerezo, 2021). Although the private sector currently predominantly applies GMMs in contained systems as bio-factories, a field of applications that we excluded, our results, gained mainly from the scientific literature, point to an increased research interest in potential environmental applications. Van der Vlugt et al. (2020) also searched the scientific literature, but focused on the experimental and marketing stage, i.e., products already tested or used in the environment, excluding basic and application-oriented research.

More recently, the EC requested a scientific opinion on new developments in biotechnology applied to microorganisms from EFSA (EC, 2022). An online survey launched by EFSA in spring of 2023 in preparation for a HS to be conducted in the course of this mandate mostly identified starter cultures, probiotics and non-purified products such as enzymes and lipids, but also biopesticides (Kagkli, 2023). Conducted in parallel to our study, this survey found seven applications of GMMs produced with NGTs to be on the market worldwide, of which only one consists of GMMs capable of propagation or transfer of genes (i.e., category 4 according to EFSA guidance, EFSA, 2011). The recently published results of the EFSA HS reveal a focus on applications as (or as a source of) food or food additives (Ballester et al., 2023). Interestingly, apart from one application of a microalgae for the production of aquatic bait with high PUFAs, only one environmental application was identified, the soil bacterium (*Klebsiella variicola*) applied as a supplement to nitrogen fertilisers (Ballester et al., 2023).

In contrast, our HS identified multiple research efforts with GMMs for use in the environment, highlighting respective applications for agricultural production in the future. Such GMMs developed for application as plant protection products or plant biostimulants in agriculture may, however, also be present in food and feed products (Mullins et al., 2022) and thus have implications for food and environmental safety.

Overall, differences in the research focus are apparent between the four groups of organisms reviewed here. For GM terrestrial animals including GM fish the emphasis lies on traits with relevance for production processes, i.e., disease resistance and performance. In GM fish this also includes modified reproductive traits. Unicellular organisms however, are modified to be used as biological agents for bioremediation, biocontrol or biofertilization and as production platforms for biological substances such as biofuels. Although we did not analyse the identified applications with respect to the various modification techniques applied and types of modifications achieved, we noticed that genome editing approaches are more frequently used in terrestrial animals and fish than in unicellular organisms, where transgenic approaches still seem to dominate. In microalgae, for example, technical hurdles exist with respect to the application of certain genome editing tools (for review see Doron et al., 2018, Jeon et al., 2017). Overall, in contrast to transgenesis, which creates gene insertions at random sites in the genome, genome editing approaches in GM terrestrial animals and fish mostly result in gene knock-outs and indels at specific sites in the genome.

### 4.3 Few market-relevant applications in the pipeline

Despite high expectations for increased numbers of GMOs caused by applications of NGTs, the overall number of market-relevant GM applications identified in our HS was rather low, i.e., nine for GM terrestrial animals and four for GM fish and GM algae and five for GMMs. Of those, four terrestrial animals, three fish, no algae and three microorganisms were developed by genome-editing approaches. A complete picture of market-relevant developments of GMOs cannot be obtained exclusively from a HS activity covering the scientific literature. The product development of any GMO usually takes years and does not necessarily result in a marketed product, like, for example, the EnviroPig™ (CBAN, 2023) or is not published in the scientific literature immediately. We therefore complemented the literature search with the screening of grey literature, such as OECD reports, websites and databases of national authorities. Publicly available information on authorisations, notifications and risk assessment reports substantially increased the overall number of identified market-relevant applications in most organism groups, except for GM animals (Figure 1).

Relevant information on GM products notified and/or authorized for deliberate release or placing on the market is available from online sources of regulatory authorities from different countries, e.g., Australia (OGTR, 2023), Canada (ECCC, 2023). However, we encountered various hurdles.

• Not all relevant information is available in English, often only in national languages (e.g., Japanese, Portuguese, Spanish).
• Typically, only limited information on the regulated products is available. Relevant information, e.g., on the specific genetic modifications and the traits of particular GM products, is considered confidential and thus not made publicly available.
• Notifications for approvals and approvals granted are not indicative of whether the respective applications are indeed released into the environment or placed on the market. A permit issued in the US in 2020 for large scale field trials involving the release of a GM virus in Orange plantations, for example, was not implemented until April 2023. The Enviropig™ for instance was tested in controlled facilities in 2009 in Canada (Environment Canada, 2012), but never developed further for use as human food (CBAN, 2023).
• Typically, regulatory information about granted authorizations does not provide any details regarding either the extent or the discontinuation of the marketing of GM products by consent holders.
• Regulatory frameworks and competences of national authorities differ considerably between countries with respect to products of NGTs, the regulation of which is still under discussion in some countries (for review see Eckerstorfer et al., 2019; Menz et al., 2020). If products of NGTs are generally not regulated based on technological criteria (e.g., Japan, Australia) or deregulated after being considered of low risk following a case-specific scientific review (e.g., in the US, Canada or Brazil), only restricted information on the product is available to the public (see for example, US FDA, 2023 a; b). Thus, information necessary to determine for example, species and traits modified, the techniques used and the molecular mechanisms established to achieve the trait, i.e., information needed to clearly identify a certain product and also the basis for risk assessment, is not always provided.

Despite these difficulties, in our view a HS profits greatly from information from national authorities to gain insight into upcoming market applications and we therefore support personal requests for information to national experts in regulatory authorities. Similarly, we think that contributions of scientific experts from academia and industry would be important for a HS.

### 4.4 Challenges of novel types of GMOs from the risk assessors’ perspective

The broad range of GM species, traits and fields of applications identified in this HS indicates a variety of challenges for the assessment of potential negative effects for the environment. An environmental risk assessment–beside information on the organism, the modified trait, and the intended use–requires information on the genetic modification of the organisms, at the genomic as well as the phenotypic level (for overview see, e.g., EFSA, 2010; EFSA, 2013; CBD, 2018). Furthermore, information on the modified species, its biology, and ecological interactions is needed. For example, the broad range of potential applications identified in this HS shows a variety of different mechanisms of action resulting in a multitude of possible pathways to harm. Some traits are brought about by gene knockouts, others by the expression of specific effector molecules like antimicrobial peptides or proteases, while others express RNAs triggering an RNAi response in the modified or in other organisms (e.g., pest species). Every mechanism of action has different implications for risk assessment and thus different applications vary in respect to their level of risk. Risk assessment experience with the new types of organisms discussed here as well as knowledge about potential pathways to harm and potential unintended effects are limited. While detailed knowledge on the species biology is certainly available for farmed animals, less information is available for unicellular organisms and their environmental interactions. While for GM animals EFSA published a first guidance document for the environmental risk assessment in 2013 (EFSA, 2013), comparable guidance for GMMs is missing.

In addition, it is crucial to consider substantial biological and ecological differences between unicellular organisms and higher animals. For example, the small size of microalgae or bacteria, the higher number of individuals involved in applications, their short generation time, their ability for horizontal gene transfer and to form resting stages as well as different pathways of spread and dispersal pose new questions and challenges for the environmental risk assessment. Furthermore, these characteristics also have consequences for practical applications. For instance, the stability of the modified trait is paramount for the intended use, but might be challenging to maintain among a large number of unicellular organisms with high replication rates. In particular, in case of intended use in the environment, e.g., in case of biofertilizers, but also if spread occurs into natural habitats, the genetic and phenotypic stability of these organisms under variable environmental conditions is questionable with unknown implications for natural ecosystems. Compared to GM microalgae and GMMs, the reproduction of GM terrestrial animals identified in this HS is generally subject to human control and horizontal gene transfer is not a risk assessment issue.

Furthermore, GMMs to be applied in mixtures or used to fulfill multiple purposes, for example, biocontrol agents that strengthen plant health and growth characteristics, i.e., by acting as biofertilizers, would lead to particular challenges. At present, even applications with unmodified micoorganisms in the environment using natural or artificial communities of microorganisms (also called synthetic microbial consortia, also known by the name effective microorganisms) targeting the alteration of microbiomes for specific purposes (e.g., in the soil to enhance fertility or bind toxic pollutants) at larger scales are only in their infancy, with limited experience on interactions between MOs and on microbial communities. In the future soil microbiome engineering may include genome editing of MOs and may even be based on *in situ* simultaneous genetic modification of several microorganism species (also called community gene editing) (Jansson et al., 2023). Together with obvious challenges to maintain the stability of microbial consortia in the environment (Grandel et al., 2021), this would substantially increase the complexity of risk assessments.

In addition to environmental effects, the genetic modification of animals can also impact animal health and welfare (Weaver and Morris, 2005; Eriksson et al., 2018; De Graeff et al., 2019), which also have to be taken into account in the risk assessment (EFSA, 2012; Kuzma et al., 2023). The trait of main interest identified here, i.e., enhanced muscle growth, clearly has implications for animal health and welfare. Knowledge on welfare aspects and criteria for their assessment is well established for mammals and birds (Broom and Fraser, 2015), increasingly established for fish (Segner et al., 2019; Seibel et al., 2020), and also explored for GM animals, including specifically those developed with NGT (Haskell et al., 2023).

## 5 Conclusion

An increase in GMOs, in particular products of NGTs, can be expected on the market worldwide and in the EU in the near future. In order to ensure safe and responsible use of GMOs in the environment, regulatory oversight including risk assessment is necessary, and considerable regulatory oversight has been established worldwide. A thorough case-by-case assessment of various species-traits-environment combinations with respect to their potential environmental implications, however, depends on detailed information on the modified organism, its traits, its use and environmental interactions as well as appropriate guidance. We predict that the current limited level of experience and limited amount of available scientific information will constitute a challenge in the near future. In our view, this HS clearly underlines the urgency for risk assessors and competent authorities to prepare for respective information needs.

EFSA’s scientific opinion on new developments in biotechnology applied to microorganisms, as mandated by the EC (2022), is expected to be published in June 2024 (Kagkli, 2023). Additional EC-mandated work by EFSA is under way on a scientific opinion on new developments in biotechnology applied to animals, a draft of which is due in January 2025 (Ardizzone, 2023). We believe that the overview presented here provides valuable input for both tasks, as it can serve as a basis for the identification of assessment needs in risk assessment. Furthermore, it can help to identify potential risk issues and assist the review of existing guidance documents included in the EC mandate (EC, 2022). Further work, however, e.g., regarding potential environmental effects, will be needed in order to assist a thorough review of existing risk assessment guidance on national, EU and international levels.

## Data Availability

The original contributions presented in the study are included in the article/Supplementary Material, further inquiries can be directed to the corresponding author.
