# Supplementary material for: Horizon scanning of potential environmental applications of terrestrial animals, fish, algae and microorganisms produced by genetic modification, including the use of new genomic techniques
Source: Front Genome Ed. 2024 Jun 13;6:1376927. doi: 10.3389/fgeed.2024.1376927 (PMC11208717; doi:10.3389/fgeed.2024.1376927)
Supplement: Supplementary file 1 [file Table1.DOCX]

Supplementary Material

Supplementary Table 1: Keywords and synonyms used in the literature search on GM animals; asterisks indicate wild cards for various endings

| **category** | **general keyword category or filter category** | **keywords and synonyms** |
| --- | --- | --- |
| intervention | genetic modification | “genetic* modifi*”, “gen* edit*”, “recombin*”, “genetic* engineer*”, “transgen*”, “gen* silenc*” |
| organism | animal | “animal”, “livestock”, “pets”, “ruminants”, “cattle”, “sheep”, “goats”, “pig”, “swine”, “poultry”, “chicken”, “quail” |
| trait | disease control | “disease resistance”, “genetic diseases”, “virus resistance”, “African Swine Fever (ASF) resistance”, “Avian influenza resistance”, “Bovine respiratory disease (BRD) resistance”, “Porcine Reproductive and Respiratory Syndrome (PRRS) resistance”, “tuberculosis resistance”, “resilience”, “lysostaphin” |
|  | performance | “muscle growth”, “myostatin”, “wool growth”, “wool length”, “wool production” |
|  | reproduction | “all-female”, “all-male”, “female phenotype”, “male infertility”, “male phenotype”, “sex control”, “sex reversal”, “sexing”, “SRY gene”, “Y chromosome” |
|  | product quality | “allergy”, “boar taint”, “egg composition”, “fat-1“, “human lactoferrin”, ”PUFAs”, “omega 3”, “omega 6”, “wool quality”, “β-lactoglobulin” |
|  | welfare | “stress tolerance”, “castration”, “dehorning”, “hornlessness”, “polledness”, “diluted coat”, “heat tolerance”, “slick gene”, “cold tolerance”, “thermoregulation” |
| field of application | application | “agriculture”, “agronomic traits”, “husbandry”, “livestock”, “farm”, “precision breeding”, “new breeding method”, “agricultural biotechnology”, “risk assessment”, “biosafety”, “nature conservation”, “nature protection” |
| filters | keywords in search string | AND NOT “fish”, “insects”, “shrimp”, “plants”, “vaccine”, “sexed semen”, “aquaculture” |
|  | publication year | ˃ 2011 |
|  | document type | LIMIT TO “article”, “review”, “conference paper” |
|  | language | LIMIT TO “English” |
|  | subject area | EXCLUDE “medicine” |
|  | keywords of article | EXCLUDE EXACT KEYWORD “mouse”, “mice” |

Supplementary Table 2: Keywords and synonyms used in the literature search on GM fish; asterisks indicate wild cards for various endings

| **category** | **general keyword category or filter category** | **‘synonyms’** |
| --- | --- | --- |
| intervention | genetic modification | “genetic* modifi*”, “gen* edit*”, “recombin*”, “genetic* engineer*”, “transgen*”, “gen* silenc*” |
| organism | fish | “fish”. “salmon”, “carp”, “tilapia”, “catfish” |
| trait | disease control | “abiotic stress”, “disease reduction”, “disease resistance”, “lice resistance”, “parasite resistance”, “reovirus resistance”, “virus resistance”, “stress tolerance” |
|  | performance & quality | “fat-1”, “feed conversion efficiency”, “muscle development”, ”muscle growth”, “myostatin”, “n-3 PUFA” |
|  | reproduction | “all-female”, “all-male”, “germline transmission”, “sex control”, “sex reversal”, “sterility” |
|  | pigmentation | “coloration”, “melanin”, “pigmentation”, “skin color” |
| field of application | application | “agriculture”, “aquaculture”, “biotechnology”, “precision breeding”, “risk assessment”, “biosafety”, “nature conservation”, “nature protection” |
| filters | keywords in search string | AND NOT “plants”, “microalgae”, “vaccine” |
|  | publication year | ˃ 2011 |
|  | document type | LIMIT TO “article”, “review”, “conference paper” |
|  | language | LIMIT TO “English” |
|  | subject area | EXCLUDE “medicine” |

Supplementary Table 3: Keywords and synonyms used in the literature search on GM algae; asterisks indicate wild cards for various endings

| **category** | **general key word category  or filter category** | **‘synonyms’** |
| --- | --- | --- |
| intervention | genetic modification | "genetic* modif*”, “gen* edited”, “GM”, “GE”, “recombin*”, “genetic* engineer*”, “transgen*, “gen* silenc*” |
| organism | algae | “algae”, “micro-algae”, “seaweed” |
| field of application | environment | “environment”, “agriculture”, “aquaculture” |
|  | biotechnology | “biotechnology”, “bioengineering”, “agricultural biotechnology”, “synthetic biology” |
| general | biosafety | “biosafety”, “risk assessment”, “safety”, “risks” |
| filters | publication year | ˃ 2011 |
|  | document type | LIMIT TO “article”, “review”, “conference paper” |
|  | language | LIMIT TO “English” |
|  | subject area | EXCLUDE “medicine”, “chemistry”, “pharmacy” |

Supplementary Table 4: Keywords and synonyms used in the literature search on GM microorganisms; asterisks indicate wild cards for various endings

| **category** | **general key word category or filter category** | **‘synonyms’** |
| --- | --- | --- |
| intervention | genetic modification | “genetic* modif*”, “gen* edited”, “GM”, “GE”, “recombin*”, “genetic* engineer*”, “transgen*, “gen* silenc*”, “gen* transform*” |
| organism | microorganism | “microorganism”, “bacteri*”, “microbe”, “protozoa”, “yeast”, “fung*”, “symbiont”, “cyanobacter*” |
| field of application | environment | “environment”, “agricultur*”, “insect symbiont engineering”, “bio-remediation”, “cleaning”, “biocontrol”, “biofertilizer”, “nutraceutial*” |
| general | biosafety | “biosafety”, “safety”, “synthetic biology”, “risk assessment” |
| filters | keywords in search string | AND NOT “contained use”, “bioreactor” |
|  | publication year | ˃ 2011 |
|  | document type | LIMIT TO “article”, “review”, “conference paper” |
|  | language | LIMIT TO “English” |
|  | subject area | EXCLUDE “medicine”, “chemistry”, “pharmacy” |
