# Supplementary material for: Horizon scanning of potential environmental applications of terrestrial animals, fish, algae and microorganisms produced by genetic modification, including the use of new genomic techniques
Source: Front Genome Ed. 2024 Jun 13;6:1376927. doi: 10.3389/fgeed.2024.1376927 (PMC11208717; doi:10.3389/fgeed.2024.1376927)
Supplement: Supplementary file 2 [file Table2.docx]

Supplementary Material

Supplementary Table 5: Applications of GM cattle and GM buffalo in basic research

| **Modified organism** | **Field of Application** | **Purpose of Development / Intended Trait** | **References** |
| --- | --- | --- | --- |
| Cattle | Animal welfare | Hornlessness | Tan et al., 2013; Loretts O. et al., 2020a |
|  | Disease control | Correction of the Isoleucyl-tRNA synthetase (IARS) syndrome | Ikeda et al., 2017 (cited in Wani et al., 2023) |
|  |  | Resistance to Bovine respiratory disease (BRD / *M. haemolytica*) | Shanthalingam et al., 2016 (cited in Wani et al., 2023) |
|  |  | Resistance to Bovine leukemia virus (BLV) | Loretts O. et al., 2020b |
|  |  | Resistance to brucellosis | Karponi et al., 2019 (Singh and Ali, 2021) |
|  |  | Resistance to Johne´s disease | Mallikarjunappa et al., 2020 (cited in Singh and Ali, 2021) |
|  |  | Resistance to pestiviruses | Isken et al., 2019 |
|  |  | Resistance to prion diseases | Bevacqua et al., 2016 (cited in Wang et al., 2022); K.-E. Park et al., 2020 |
|  | Performance | Enhanced (muscle) growth | Proudfoot et al., 2015 |
|  | Product quality | Elimination of β-lactoglobulin in milk | Wei et al., 2015; Koloskova et al., 2021 (cited in Raza et al., 2022) |
|  |  | With human β-defensin-3 enriched milk | Yu et al., 2013 (cited in van der Berg et al., 2020) |
|  | Reproduction | Male to female sex reversal | Xi et al., 2019; Wang et al., 2021 |
| Buffalo | Reproduction | Pre-determination of sex | Zhao et al., 2020 (cited in Singh and Ali, 2021) |

Supplementary Table 6: Applications of GM cattle in application-oriented research

| **Modified organism** | **Field of Application** | **Purpose of Development / Intended Trait** | **References** |
| --- | --- | --- | --- |
| Cattle | Animal welfare | Hornlessness | Carlson et al., 2016; Schuster et al., 2020 (cited in Singh and Ali, 2021) |
|  |  | Heat tolerance | Porto-Neto et al., 2018 (cited in van Eenennaam, 2019); Hansen, 2020 (cited in Menchaca, 2021) |
|  | Disease control | Resistance to foot and mouth disease virus (FMDV) | Yu et al., 2014 |
|  |  | Resistance to mastitis (*Staphylococcus aureus*) | Liu et al., 2013; X. Liu et al., 2014 |
|  |  | Resistance to tuberculosis (*M. bovis*) | Wu et al., 2015 (cited in Wani et al., 2023); Gao et al., 2017 |
|  | Performance | Enhanced (muscle) growth | Luo et al., 2014 (cited in Wani et al., 2023); Gim et al., 2022a; Gim et al., 2022b (cited in Wani et al., 2023) |
|  | Product quality | Elimination of β-lactoglobulin in milk | Sun et al., 2018 (cited in Wani et al., 2023); Wei et al., 2018 (cited in Menchaca, 2021) |
|  |  | Low lactose content in milk | Su et al., 2018 (cited in Wani et al., 2023) |
|  |  | Higher n-3 PUFA content in milk | Wu et al., 2012 (cited in van der Berg et al., 2020) |
|  |  | Higher n-3 PUFA content in meat | Wu et al., 2012 (cited in Fernando and Duran, 2017); Cheng et al., 2015 |
|  | Reproduction | Female to male sex reversal | Owen et al., 2021 (cited in Menchaca, 2021) |
|  |  | Sterile surrogates | Ideta et al., 2016 (cited in Menchaca, 2021); Ciccarelli et al., 2020 (cited in Menchaca, 2021 |

Supplementary Table 7: Applications of GM small ruminants in basic research

| **Modified organism** | **Field of Application** | **Purpose of Development / Intended Trait** | **References** |
| --- | --- | --- | --- |
| Goat | Performance | Enhanced (muscle) growth | Lu et al., 2013; Tan et al., 2013; Tripathi et al., 2013; Patel et al., 2014; Patel et al., 2015; Proudfoot et al., 2015; X. Wang et al., 2015; Yu et al., 2016; Zhang et al., 2018; J. Zhang et al., 2019 |
|  | Product quality | Higher n-3 PUFA content in meat | Zhang et al., 2018 |
| Sheep | Disease control | Resistance to E. coli infections | Li et al., 2021 |
|  | Performance | Enhanced (muscle) growth | Tang et al., 2012; Tripathi et al., 2013; C. Liu et al., 2014; Proudfoot et al., 2015; Wang et al., 2016b; Zhang et al., 2016 (cited in Wani et al., 2023); Zhao et al., 2016 |
|  | Product quality | Higher n-3 PUFA content in meat | Luo et al., 2020 |

Supplementary Table 8: Applications of GM small ruminants in application-oriented research

| **Modified organism** | **Field of Application** | **Purpose of Development / Intended Trait** | **References** |
| --- | --- | --- | --- |
| Goat | Performance | Enhanced (muscle) growth | Kumar et al., 2014; Ni et al., 2014; He et al., 2018; Wang et al., 2018 |
|  |  | Enhanced fiber yield | Wang et al., 2016a (cited in Wani et al., 2023) |
|  |  | Enhanced milk yield | Zhang et al., 2014 |
|  | Product quality | Elimination of β-lactoglobulin in milk | Cui et al., 2015; Zhu et al., 2016; Zhou et al., 2017 |
|  |  | High human-lactoferrin content in milk | Cui et al., 2015 |
|  | Reproduction | Sterile surrogates | Ciccarelli et al., 2020 (cited in Menchaca, 2021) |
| Sheep | Performance | Enhanced (muscle) growth | Hu et al., 2013; Crispo et al., 2015; Li et al., 2016 (cited in Jiang and Shen, 2019); Y. Zhang et al., 2019 (cited in Yuan et al., 2020); Zhou et al., 2022 |
|  |  | Enhanced wool yield | Li et al., 2017 |
|  | Product quality | Alteration of the coat color | Zhang et al., 2017 (cited in Wani et al., 2023) |

Supplementary Table 9: Applications of GM pigs in basic research

| **Field of Application** | **Purpose of Development / Intended Trait** | **References** |
| --- | --- | --- |
| Disease control | Resistance to African swine fever (ASF) | Lillico et al., 2013 (cited in Wang et al., 2022); Tan et al., 2013; Hübner et al., 2018 (cited in Liu et al., 2022) |
|  | Resistance to classical swine fever (CSF) | Yan et al., 2014 |
|  | Resistance to porcine deltacoronavirus (PDCoV) | Zhu et al., 2018 (cited in Liu et al., 2022) |
|  | Resistance to porcine reproductive and respiratory syndrome (PRRS) | Whitworth et al., 2014 (cited in Tu et al., 2022) |
|  | Resistance to pseudorabies virus (PRV) | Tang et al., 2017 (cited in Liu et al., 2022) |
| Performance | Enhanced (muscle) growth | Zou et al., 2019 (cited in Liu et al., 2022); X. Li et al., 2020; Wei et al., 2020 (cited in Wani et al., 2023); Pan et al., 2021 |
| Product quality | α-gal (Galactose-alpha-1,3-galactose) free meat | Hauschild et al., 2011^^[[1]](#footnote-1)^^ |

Supplementary Table 10: Applications of GM pigs in application-oriented research

| **Field of Application** | **Purpose of Development / Intended Trait** | **References** |
| --- | --- | --- |
| Animal welfare | Cold tolerance | Zheng et al., 2017 (cited in Liu et al., 2022) |
|  | No castration (due to delayed adolescence) | Flórez et al., 2022^^[[2]](#footnote-2)^^ |
| Disease control | Resistance to African swine fever (ASF) | Lillico et al., 2016; McCleary et al., 2020 (cited in Wang et al., 2022) |
|  | Resistance to classical swine fever (CSF) | Xie et al., 2018 (cited in Wang et al., 2022); Xie et al., 2020 (cited in Wani et al., 2023) |
|  | Resistance to foot and mouth disease virus (FMDV) | Hu et al., 2015 (cited in Liu et al., 2022) |
|  | Resistance to hepatitis E virus (HEV) | Yugo et al., 2018 (cited in Wang et al., 2022) |
|  | Resistance to porcine contagious pleuropneumonia  (*Actinobacillus pleuropneumoniae*) | Yang et al., 2015 (cited in Y.-F. Wang et al., 2017) |
|  | Resistance to porcine deltacoronavirus (PDCoV) | K. Xu et al., 2020 |
|  | Resistance to porcine epidemic diarrhea virus (PEDV) | Tu et al., 2019 (cited in Wang et al., 2022) |
|  | Resistance to porcine reproductive and respiratory syndrome (PRRS) | Whitworth et al., 2016 (cited in Wani et al., 2023); Burkard et al., 2017; Lu et al., 2017 (cited in Liu et al., 2022); Wells et al., 2017 (cited in Wani et al., 2023); Whitworth and Prather, 2017 (cited in Tu et al., 2022); Burkard et al., 2018; Yang et al., 2018 (cited in Wang et al., 2022); Chen et al., 2019 (cited in Wani et al., 2023); Guo et al., 2019 (cited in Wang et al., 2022); Wang et al., 2019 (cited in Liu et al., 2022); K. Xu et al., 2020; Tanihara et al., 2021 (Tu et al., 2022) |
|  | Resistance to pseudorabies virus (PRV) | Xie et al., 2020 (cited in Wani et al., 2023) |
|  | Resistance to transmissible gastroenteritis virus (TGEV) | Luo et al., 2019 (cited in Liu et al., 2022); Whitworth et al., 2019 (cited in Wang et al., 2022); K. Xu et al., 2020 |
|  | Enhanced antibacterial activity of sow milk | Han et al., 2020 (cited in Wani et al., 2023) |
| Performance | Enhanced (muscle) growth | Qian et al., 2015 (cited in Wani et al., 2023); K. Wang et al., 2015; Bi et al., 2016; Rao et al., 2016^^[[3]](#footnote-3)^^, K. Wang et al., 2017 (cited in Tu et al., 2022); Xiang et al., 2018 (cited in Liu et al., 2022); Zou et al., 2018 (cited in Tu et al., 2022); Liu et al., 2019 (cited in Wani et al., 2023); Bi et al., 2020 (cited in Wani et al., 2023); R. Li et al., 2020 (cited in Tu et al., 2022); Fan et al., 2022 |
|  | Increased lean meat percentage | Zheng et al., 2017 (cited in Liu et al., 2022); Bi et al., 2020 (Wani et al., 2023); |
|  | Enhanced weight gain of piglets through human α-lactalbumin in sow milk | Ma et al., 2016 (cited in Y.-F. Wang et al., 2017) |
| Product quality | Higher n-3 PUFA content in meat | Zhou et al., 2014; Li et al., 2018 (cited in Liu et al., 2022); Tang et al., 2019; You et al., 2021 (cited in Liu et al., 2022) |
|  | Increased intramuscular fat | Gu et al., 2021 (cited in Tu et al., 2022) |
| Reproduction | Improved spermatogenesis ability | Xu et al., 2016 (cited in Y.-F. Wang et al., 2017) |
|  | Male to female sex reversal | Kurtz et al., 2021 |
|  | Sterile surrogates | Park et al., 2017; Ciccarelli et al., 2020 (cited in Menchaca, 2021) |

Supplementary Table 11: Applications of GM poultry and other species in basic research

| **Modified organism** | **Field of Application** | **Purpose of Development / Intended Trait** | **References** |
| --- | --- | --- | --- |
| Chicken | Disease control | Resistance to Avian influenza virus (AIV) | Park et al., 2021 (cited in Wang et al., 2022) |
|  | Performance | Enhanced (muscle) growth | Lee et al., 2017; K. Y. Lee et al., 2020 |
|  | Product quality | Changed composition of the egg white | K. Y. Lee et al., 2020 |
| Quail | Performance | Enhanced (muscle) growth | J.-W. Park et al., 2020 |
| Horse | Disease control | Correction of unwanted mutations | Pinzon-Arteaga et al., 2020 (cited in Wani et al., 2023) |
|  | Performance | Enhanced (muscle) growth | Moro et al., 2020 |

Supplementary Table 12: Applications of GM poultry and other species in application-oriented research

| **Modified organism** | **Field of Application** | **Purpose of Development / Intended Trait** | **References** |
| --- | --- | --- | --- |
| Chicken | Disease control | Resistance to Avian leukosis virus (ALV) | Hellmich et al., 2020 (cited in Wang et al., 2022); Koslová et al., 2020 (cited in Wani et al., 2023) |
|  | Performance | Enhanced (muscle) growth | Kim et al., 2020 |
|  | Reproduction | Sex determination pre-hatch | Doran et al., 2018 |
| Quail | Performance | Enhanced (muscle) growth | J. Lee et al., 2020 (cited in Volkova et al., 2021); Lee et al., 2021 |
|  | Product quality | Changed plumage color | Lee et al., 2019 (cited in Volkova et al., 2021) |
| Rabbit | Performance | Enhanced (muscle) growth | Guo et al., 2016; Shang et al., 2022; Zheng et al., 2022 |
|  |  | Enhanced fur production | Y. Xu et al., 2020 (cited in Wani et al., 2023) |

Supplementary Table 13: Field trials and commercial use of GM animals in different countries

| **Modified organism** | **Targeted trait** | **Application/registration/approval** | **Country/year** | **Authority** | **Source/reference** |
| --- | --- | --- | --- | --- | --- |
| Cattle | Resistance to Bovine Respiratory Disease | Patent | USA/2016 | Office of commerciali-zation, Washington State University | Office of Commercialization, 2016 |
|  | Heat tolerance | RA^[[4]](#footnote-4)^ consultation | USA/2022 | FDA^[[5]](#footnote-5)^ | FDA, 2022 |
|  | Hornlessness | RA consultation | Brazil/2018 | CTN Bio^[[6]](#footnote-6)^ | CTNBio 2023 |
|  | Enhanced (muscle) growth | RA consultation | Brazil/2018 | CTN Bio^6^ | CTNBio 2023 |
|  | Expression of human growth hormone in milk | Field trials | Argentina/ since 2005 | INTA^[[7]](#footnote-7)^ | OECD, 2022 |
|  | Expression of antibodies against rotavirus in milk | Field trials | Argentina/ since 2005 | INTA^7^ | OECD, 2022 |
|  | Expression of human lysozyme and lactoferrin | Field trials | Argentina/ since 2011 | INTA^7^ | OECD, 2022 |
| Pig | Lower phosphorus excretion (EnviroPig™) | RA | Canada/2009 | Environment Canada; Health Canada | ECCC, 2009 |
|  | α-gal (Galactose-alpha-1,3-galactose) free meat | Approved for commercial sale | USA/2020 | FDA^5^ | US FDA, 2020 |

1. derived from additional literature search [↑](#footnote-ref-1)
2. derived from additional literature search [↑](#footnote-ref-2)
3. Derived from additional literature search [↑](#footnote-ref-3)
4. Risk assessment [↑](#footnote-ref-4)
5. US Food and Drug Administration [↑](#footnote-ref-5)
6. National Technical Commission of Biosafety [↑](#footnote-ref-6)
7. Instituto Nacional de Tecnología Agropecuaria [↑](#footnote-ref-7)
