# Supplementary material for: Horizon scanning of potential environmental applications of terrestrial animals, fish, algae and microorganisms produced by genetic modification, including the use of new genomic techniques
Source: Front Genome Ed. 2024 Jun 13;6:1376927. doi: 10.3389/fgeed.2024.1376927 (PMC11208717; doi:10.3389/fgeed.2024.1376927)
Supplement: Supplementary file 3 [file Table5.docx]

Supplementary Material

Supplementary Table 21: Applications of GM microorganisms in basic research

| Field of Application | Purpose of Development / Intended Trait | Modified organism | References |
| --- | --- | --- | --- |
| Biofertilizer | Increased efficiency of rhizobacterial root symbiosis | Rhizobacteria,  Hyphomicrobiales (*Sinorhizobium meliloti*) | García-Tomsig et al., 2022 |
|  | Engineered lithoheterotrophy | Chemolithoautotrophic bacteria, Mariprofundales (*Mariprofundus ferrooxydans*) | Jain and Gralnick, 2021 |
|  | Quorum sensing regulation | Enterobacteriales (*Escherichia coli*) | Pai et al., 2012 |
| Paratransgenesis | Aphid control | Gut associated bacteria,  Enterobacterales (*Serratia symbiotica*) | Elston et al., 2021 |
|  | Development of entomopathogenic nematode agents | Nematode associated bacteria, Enterobacterales (*Photorhabdus heterorhabditis*) | Lulamba et al., 2021 |
|  | Symbionts for control of different pest species via RNAi | Actinomycetales (*Rhodococcus rhodnii*); Enterobacteriales (BFo2) | Whitten et al., 2016 (cited in Whitten and Dyson, 2017) |
|  | Biocontrol agent | Intracellular bacterial symbionts, Rickettsiales (*Wolbachia pipientis*) | Fallon, 2021 |
| Biocontrol | Root-knot nematode control in potato | Transgenic soil bacteria | Bali et al., 2021 |
|  | Biological fly control agents | Bacillales (*Bacillus thuringiensis*), Ascomycota (*Beauveria bassiana*) | Geden, 2012 |
|  | Crop pest control | Entomopathogenic fungi | Méndez-González et al., 2022 |
|  | Avirulent S. enterica as research model | Enterobacterales (*Salmonella enterica serovar Typhimurium*) | Moraes et al., 2016 |
|  | Enhanced biocontrol of locusts | *Metarhizium acridum* | Peng and Xia, 2014; Peng and Xia, 2015 (cited in Pan and Zhang, 2020) |
|  | Apathogenic V. longisporum | Fungal plant pathogen (*Verticillium longisporum*) | Timpner et al., 2013 |
|  | Control of diffusible signal factor dependent bacteria | Xanthomonadales (*Xanthomonas campestris*) | Wang et al., 2020 |
|  | Genetically recoded bacterium to support biocontainment | Enterobacteriales (*Escherichia coli*) | Rovner et al., 2015 |
|  | pH and temperature-dependent biocontainment system | Enterobacteriales (*Escherichia coli*) | Stirling et al., 2020 |
| Bioremediation | Enhanced formation of biofilms for 1-chlorobutane degradation | Pseudomonadales (*Pseudomonas putida KT2440*) | Benedetti et al., 2016 (cited in Nwankwegu et al., 2022) |
|  | Conversion of hemicellulose and chitin hydrolysates into lipids | Oleaginous yeast, Basidomycota (*Trichosporon oleaginosus*) | Görner et al., 2016 |
|  | Mercury bioremediation through volatilization | Mercury-resistant bacteria | Priyadarshanee et al., 2022 |
|  | Development of bacteria with ability to degrade ephedrine isomers | Micrococcales (*Arthrobacter* sp.) | Shanati and Ansorge-Schumacher, 2020 |
|  | Modified laccase for enhanced dye decolorisation | Enterobacteriales (*Escherichia coli*) | Wang et al., 2017 (cited in Patel et al., 2022) |
|  | Microbial consortia with GM fungi for better degradation of polycyclic aromatic hydrocarbons | Ascomycota (*Aspergillus niger*) | Zafra et al., 2017 (cited in Sakshi and Haritash, 2020) |
|  | Cadmium accumulation | Ascomycota (*Saccharomyces cerevisiae*) | Zhang et al., 2021 |
|  | Expression of surface-displayed ChrB for removal of Cr(VI) | Enterobacteriales (*Escherichia coli*) | Zhou et al., 2020 |

Supplementary Table 22: Applications of GM microorganisms in application-oriented research on bioremediation

| Field of Application | Purpose of Development / Intended Trait | Modified organism | References |
| --- | --- | --- | --- |
| Bioremediation | Remediation of soils contaminated with polycyclic aromatic hydrocarbons | Pseudomonadales (*Pseudomonas aeruginosa*) | Cao et al., 2012 |
|  | Enhanced arsenic methylation and volatilization | Pseudomonadales (*Pseudomonas putida*) | Chen et al., 2014 |
|  | Degradation of a mixture of benzene, toluene, ethylbenzene, and xylenes | Pseudomonadales (*Pseudomonas putida*) | Chicca et al., 2020 (cited in Kumar et al., 2022) |
|  | Increased nitrobenzene degradation | Enterobacteriales (*Escherichia coli*) | Deng et al., 2022 |
|  | Enhanced degradation of 2,4-dinitrotoluene | Cyanobacteria (*Synechococcus elongatus*), Pseudomonadales (*Pseudomonas putida*) | Fedeson et al., 2020 |
|  | Enhanced degradation of pesticides (chlorpyrifos and carbofuran) | Pseudomonadales (*Pseudomonas putida*) | Gong et al., 2016 |
|  | Simultaneously degradation of organophosphates, pyrethroids and carbamates | Pseudomonadales (*Pseudomonas putida*) | Gong et al., 2018 (cited in Zhao et al., 2021) |
|  | Enhanced arsenic methylation and volatilization | Bacillales (*Bacillus subtilis*) | Huang et al., 2015 |
|  | Enhanced degradation of para-nitrophenol | Pseudomonadales (*Pseudomonas putida*) | Huo et al., 2022 |
|  | Biocontainment of a toluene-degrading microbe | Pseudomonadales (*Acinetobacter sp*.) | Ishikawa et al., 2021 |
|  | Biofloc-forming cells for improved phenol removal | Enterobacteriales (*Escherichia coli*) | Jia et al., 2020 |
|  | Removal of phosphate, nitrate, ammonium and nitrite from shrimp farming wastewater | Cyanobacteria (*Synechocystis*) | Krasaesueb et al., 2023 |
|  | Enhanced degradation of parathion and γ-hexachlorocyclohexane in a bacterium engineered for biocontainment | Sphingomonadales (*Sphingomonas paucimobilis*) | Lan et al., 2014 |
|  | Enhanced degradation of organophosphorus pesticide residues in the environment | Enterobacteriales (*Escherichia coli*) | Li et al., 2014 |
|  | Enhanced degradation of erythromycin at bacterial surfaces | Enterobacteriales (*Escherichia coli*) | Liu et al., 2020 |
|  | Enhanced accumulation of arsenic in bacteria | Enterobacteriales (*Escherichia coli*) | Maleki and Shahpiri, 2022 |
|  | Enhanced biodegradation of oil in spiked soil | Pseudomonadales (*Pseudomonas putida)* | Mardani et al., 2017 (cited in Sakshi and Haritash, 2020) |
|  | Enhanced arsenic volatilization in rice cultures by growth promoting yeast | Ascomycota (*Saccharomyces cerevisiae*) | Verma et al., 2019 |
|  | Enhanced degradation of phenol in wastewater | Enterobacteriales (*Escherichia coli*) | Wang et al., 2019 |
|  | Enhanced degradation of methyl-parathion | Enterobacteriales (*Escherichia coli*) | Xu et al., 2022 |
|  | Enhanced adsorption of Cadmium and degradation of parathion and γ-hexachlorocyclohexane | Sphingomonadales (*Sphingobium japonicum*) | Yang et al., 2016 |
|  | Enhanced degradation of organochlorine and organophosphate pesticides | Enterobacteriales (*Escherichia coli*) | Yang et al., 2012 (cited in Bilal and Iqbal, 2020) |
|  | Enhanced degradation of different pesticides for in situ remediation | Pseudomonadales (*Pseudomonas putida*) | Yi et al., 2016 |
|  | Enhanced degradation of nicotine in tobacco waste | Pseudomonadales (*Pseudomonas sp.)* | Zhang et al., 2019 |
|  | Enhanced degradation of phenanthrene | Pseudomonadales (*Pseudomonas sp.)* | Zhou et al., 2013 |

Supplementary Table 23: Applications of GM microorganisms in application-oriented research on biocontrol

| Field of Application | Purpose of Development / Intended Trait | Modified organism | References |
| --- | --- | --- | --- |
| Biocontrol | miRNA producing biocontrol agent | Ascomycota (*Beauveria bassiana*) | Asgari, 2023 |
|  | Biocontrol of wine-related yeasts through overexpression of saccharomycin | Ascomycota (*Saccharomyces cerevisiae*) | Branco et al., 2019 |
|  | Biocontrol of plant-parasitic nematodes by expression of dsRNA | Ascomycota (*Botrytis cinerea*) | Ding et al., 2021 |
|  | Biocontrol agent with higher antifungal activity against poplar canker disease | Burkholderiales (*Burkholderia pyrrocinia*) | He et al., 2018 |
|  | Biocontrol of whiteflies by expression of dsRNA | Ascomycota *(Isaria fumosorosea*) | Hu and Wu, 2016 |
|  | Enhanced antifungal activity in plant growth-promoting rhizobacterium | Pseudomonadales (*Pseudomonas protegens*) | Jing et al., 2020 |
|  | Enhanced biocontrol of agricultural insect pests | Ascomycota (*Beauveria bassiana*) | Kim et al., 2013 |
|  | Probiotic effect of bacteria with increased spore forming ability | Eubacteriales (*Clostridium tyrobutyricum*) | Liang et al., 2020 |
|  | Enhanced antimalarial effect mediated by a transgenic toxin (Hybrid) | Ascomycota (*Metarhizium pingshaense*) | Lovett and St Leger, 2018 (cited in Shen et al., 2020) |
|  | Biocontainment based on phosphite dependency | Cyanobacteria (*Synechococcus elongatus*) | Motomura et al., 2018 |
|  | Bacteria-mediated RNAi for control of fungal pathogens | Enterobacteriales (*Escherichia coli*) | Niño-Sánchez et al., 2021 |
|  | Increased activity of entomopathogenic fungus against locusts | Ascomycota (*Metarhizium acridum*) | Tong et al., 2021 |
|  | Bacteria-mediated RNAi for control of beet armyworm | Enterobacteriales (*Escherichia coli*) | Vatanparast and Kim, 2017 |
|  | Enhanced efficiency of Trichoderma biocontrol against Botrytis cinerea | Ascomycota (*Trichoderma harzianum*) | Xia et al., 2018 |
|  | Bacteria-mediated RNAi for control of cotton bollworm | Enterobacteriales (*Escherichia coli*) | Xiong et al., 2013 |
|  | Subunit vaccine against White Spot Syndrome Virus in shrimp | Cyanobacteria (*Synechococcus*) | Xu et al., 2021 |
|  | Enhanced colonization of plant-growth-promoting rhizobacterium increases biocontrol ability | Bacillales (*Bacillus velezensis*) | Xu et al., 2019 |
|  | Increased antifungal activity against Alternaria by expression of Cecropin A | Ascomycota (*Pichia pastoris*) | Zhang et al., 2018 |

Supplementary Table 24: Applications of GM microorganisms in application-oriented research on paratransgenesis and biofertilization

| Field of Application | Purpose of Development / Intended Trait | Modified organism | References |
| --- | --- | --- | --- |
| Paratransgenesis | Paratransgenic control of Glassy Winged Sharpshooter (*Homalodisca vitripennis)* | Enterobacteriales (*Pantoea agglomerans*) | Arora et al., 2018 |
|  | siRNA producing yeast for biocontrol of mosquito larvae | Ascomycota (*Saccharomyces cerevisiae*) | Mysore et al., 2017 |
|  | Malaria control with GM symbiotic bacteria | Enterobacteriales (*Serratia AS1*) | Wang and Zou, 2019 |
| Biofertilizer | Enhanced ammonium release | Pseudomonadales (*Azotobacter vinelandii*) | Ambrosio and Curatti, 2021 |
|  | Increased resistance against abiotic stress | Cyanobacteria  Nostocales (*Anabaena* sp.) | Chaurasia et al., 2017 |
|  | Increased nitrogen fixation ability | Pseudomonadales (*Azotobacter vinelandii*) | Das, 2019 |

Supplementary Table 25: Applications of GM microorganisms on the market and in market-development

| Field of Application | Purpose of Development / Intended Trait | Modified organism | References |
| --- | --- | --- | --- |
| Bioremediation | Naphthalene degradation with coincident bioluminescent response | *Pseudomonas fluorescens* | Sayler and Ripp, 2000 |
|  | Atrazine herbicide degradation, kilAtelAB tellurite resistance | *Pseudomonas putida* *CR30RNSLL (pADPTel)* | ECCC 2016 |
| Biocontrol | Suppression of *Salmonella* sp | *Escherichia coli* | CTN Bio, 2023 |
| Biofertilizer | Ammonium fixation | *Klebsiella variícola* | CTN Bio, 2023 |
|  | Expression of β-1,4 endoglucanase from *Bacillus subtilis* for soil conditioning | *Bacillus thuringiensis israelenses* | CTN Bio, 2023 |
