# Supplementary material for: Horizon scanning of potential environmental applications of terrestrial animals, fish, algae and microorganisms produced by genetic modification, including the use of new genomic techniques
Source: Front Genome Ed. 2024 Jun 13;6:1376927. doi: 10.3389/fgeed.2024.1376927 (PMC11208717; doi:10.3389/fgeed.2024.1376927)
Supplement: Supplementary file 4 [file Table3.docx]

Supplementary Material

Supplementary Table 14: Applications of GM fish in basic research

| **Field of Application** | **Purpose of Development / Intended Trait** | **Modified organism** | **References** |
| --- | --- | --- | --- |
| Disease control | Resistance to viral nervous necrosis (VNN) disease | Asian sea bass (*Lates calcarifer*) | Yang et al., 2021 |
|  | Viral resistance | Chinook salmon (*Oncorhynchus tshawytscha*) | Dehler et al., 2019 (cited in Blix et al., 2021) |
|  | Disease resistance | Rohu carp (*Labeo rohita*) | Chakrapani et al., 2016 (cited in Blix et al., 2021) |
| Performance | Enhanced (muscle) growth | Blotched snakehead (*Channa maculata*) | Ou et al., 2023 |
|  |  | Large yellow croaker (*Larimichthys crocea*) | Yan et al., 2022 |
|  |  | Spotted rose snapper (*Lutjanus guttatus*) | Torres-Velarde et al., 2020 |
|  |  | Yellow catfish (*Pelteobagrus fulvidraco*) | Dong et al., 2014 (cited in Lu et al., 2021) |
|  |  | Zebrafish (*Danio rerio*) | Godino-Gimeno et al., 2020 |
|  | Higher feed efficiency | Zebrafish (*Danio rerio*) | Godino-Gimeno et al., 2020 |
|  | Slow swimming behaviour | Pacific bluefin tuna  (*Thunnus orientalis*) | Higuchi et al., 2019 |
| Product quality | Loss of intermuscular bones | Zebrafish (*Danio rerio*) | Nie et al., 2021 (cited in Gui et al., 2022) |
| Reproduction | Sterility | Atlantic salmon (*Salmo salar* L.) | Wargelius et al., 2016 |
|  |  | Common carp (*Cyprinus carpio*) | Su et al., 2014 |
|  |  | Channel catfish (*Ictalurus punctatus*) | Qin et al., 2016 |
|  |  | Nile tilapia (*Oreochromis niloticus*) | Li et al., 2014 (cited in Blix et al., 2021); Chen et al., 2017 (cited in Blix et al., 2021); Tao et al., 2020 (cited in Blix et al., 2021) |
|  |  | Sterlet (*Acipenser ruthenus*) | Baloch et al., 2019 (cited in Blix et al., 2021) |
|  |  | Zebrafish (*Danio rerio*) | Noble et al., 2019 |
|  | Female to male sex reversal | Nile tilapia (*Oreochromis niloticus*) | Li et al., 2013; Xie et al., 2016 (cited in Blix et al., 2021) |
|  |  | Yellow catfish (*Pelteobagrus fulvidraco*) | Dan et al., 2018 |
| Pigmentation | Loss of pigmentation | Atlantic salmon (*Salmo salar L*.) | Edvardsen et al., 2014 (cited in Blix et al., 2021) |
|  |  | Zebrafish (*Danio rerio*) | Irion et al., 2014 (cited in Luo et al., 2021) |
|  | Red phenotype | Nile tilapia (*Oreochromis niloticus*) | Wang et al., 2023 |
|  |  | Yellow river carp (*Cyprinus carpio Haematopterus*) | Jiang et al., 2022 |

Supplementary Table 15: Applications of GM fish for disease control in application-oriented research

| **Field of Application** | **Purpose of Development / Intended Trait** | **Modified organism** | **References** |
| --- | --- | --- | --- |
| Disease control | Resistance to enteric septicemia of catfish (*Edwardsiella ictaluri*) | Channel catfish (*Ictalurus punctatus*) | Abass et al., 2022; Coogan et al., 2022 |
|  | Resistance to hemorrhagic disease of grass carp (*Grass carp reovirus*) | Grass carp (*Ctenopharyngodon idellus*) | Ma et al., 2018 |
|  | Resistance to *Vibrio vulnificus* infection | Nile tilapia (*Oreochromis niloticus*) | Chiang et al., 2020 |
|  | Resistance to furunculosis (*Aeromonas salmonicida*) | Rainbow trout (*Oncorhynchus mykiss*) | Lo et al., 2014; Chiou et al., 2014 (cited in Elaswad and Dunham, 2018) |
|  | Resistance to *Vibrio alginolyticus* infection | Zebrafish (*Danio rerio*) | Wang et al., 2014 |
|  | Higher liver bacteriolysis activity (lysozyme) | Zebrafish (*Danio rerio*) | Chengfei et al., 2017 |

Supplementary Table 16: Applications of GM fish with enhanced performance and product quality in application-oriented research

| **Field of Application** | **Purpose of Development / Intended Trait** | **Modified organism** | **References** |
| --- | --- | --- | --- |
| Performance | Enhanced (muscle) growth | Atlantic salmon (*Salmo salar* L.) | Tibbetts et al., 2013 |
|  |  | Blunt snout bream (*Megalobrama Amblycephala*) | Jiang et al., 2017; Sun et al., 2020 |
|  |  | Channel catfish (*Ictalurus punctatus*) | Khalil et al., 2017 (cited in Roy et al., 2022)  Coogan et al., 2022 |
|  |  | Common carp (*Cyprinus carpio*) | Zhong et al., 2016 |
|  |  | Gibel carp (*Carassius gibelio*) | Huang et al., 2021 |
|  |  | Mud loach (*Misgurnus anguillicaudatus*) | Tao et al., 2021 |
|  |  | Nile tilapia (*Oreochromis niloticus*) | Wu et al., 2023 |
|  |  | Olive flounder (*Paralichthys olivaceus*) | Kim et al., 2019 (cited in Roy et al., 2022) |
|  |  | Red carp (*Cyprinus carpio*) | Zhang et al., 2021 |
|  |  | Red sea bream (*Pagrus major*) | Kishimoto et al., 2018; Ohama et al., 2020; Washio et al., 2021 |
|  |  | Yellow catfish (*Pelteobagrus fulvidraco*) | Zhang et al., 2020 |
|  |  | Zebrafish (*Danio rerio*) | Silva et al., 2015 |
|  | Higher feed efficiency | Atlantic salmon (*Salmo salar* L.) | Tibbetts et al., 2013 |
|  |  | Gibel carp (*Carassius gibelio*) | Huang et al., 2021 |
|  |  | Nile tilapia (*Oreochromis niloticus*) | Wu et al., 2023 |
|  |  | Red sea bream (*Pagrus major*) | Ohama et al., 2020; Washio et al., 2021 |
|  | Higher nitrogen retention efficiency | Atlantic salmon (*Salmo salar* L.) | Tibbetts et al., 2013 |
|  | Higher lipid accumulation | Mud loach (*Misgurnus anguillicaudatus*) | Tao et al., 2021 |
| Product quality | Increased n-3 PUFA content | Atlantic salmon (*Salmo salar* L.) | Datsomor et al., 2019 (cited in Blix et al., 2021) |
|  |  | Channel catfish (*Ictalurus punctatus*) | Xing et al., 2022; Xing et al., 2023 |
|  |  | Zebrafish (*Danio rerio*) | Pang et al., 2014 |

Supplementary Table 17: Applications of GM fish with reproductive traits in application-oriented research

| **Field of Application** | **Purpose of Development / Intended Trait** | **Modified organism** | **References** |
| --- | --- | --- | --- |
| Reproduction | Sterility | Atlantic salmon (*Salmo salar* L.) | Güralp et al., 2020 (cited in Blix et al., 2021) |
|  |  | Zebrafish (*Danio rerio*) | Yan et al., 2017 (cited in Lu et al., 2021); Zhou et al., 2018 |
|  | Male to female sex reversal | Common carp (*Cyprinus carpio*) | Zhai et al., 2022 |
|  |  | Medaka (*Oryzias latipes*) | Luo et al., 2015 (cited in Lu et al., 2021) |
|  | Female to male sex reversal | Zebrafish (*Danio rerio*) | Zhang et al., 2015 (cited in Xu et al., 2023); Dranow et al., 2016 (cited in Lu et al., 2021); Lau et al., 2016 (cited in Lu et al., 2021); Zhou et al., 2018 |

Supplementary Table 18: Applications of GM fish with pigmentation traits in application-oriented research

| **Field of Application** | **Purpose of Development / Intended Trait** | **Modified organism** | **References** |
| --- | --- | --- | --- |
| Pigmentation | Loss of pigmentation | Common carp (Cyprinus carpio) | Chen et al., 2019 (cited in Blix et al., 2021); Mandal et al., 2020 (cited in Blix et al., 2021) |
|  |  | Large-scale loach (Paramisgurnus dabryanus) | Xu et al., 2019 |
|  |  | Medaka (Oryzias latipes) | Fang et al., 2018 (cited in Lu et al., 2021) |
|  |  | White crucian carp (Carassius cuvieri) | Liu et al., 2019 (cited in Blix et al., 2021) |
|  | Silver-white phenotype | Nile tilapia (Oreochromis niloticus) | Wang et al., 2022 |

Supplementary Table 19: Commercial use of GM fish in different countries

| **Modified organism** | **Targeted trait** | **Application/registration/Approval** | **Country/year** | **Authority** | **References** |
| --- | --- | --- | --- | --- | --- |
| Atlantic salmon (*Salmo salar* L.) | Enhanced (muscle) growth | Approved for commercial sale | USA/2015 | FDA^[[1]](#footnote-1)^ | FDA, 2023 |
|  |  | RA^[[2]](#footnote-2)^ | Canada/2016 | Health Canada, CFIA^[[3]](#footnote-3)^ | Health Canada, 2016 |
|  |  |  | Brazil/2021 | CTN Bio^[[4]](#footnote-4)^ | OECD, 2022 |
| Red Sea Bream (*Pagrus major*) | Enhanced (muscle) growth, higher feed efficiency | Approved for commercial sale | Japan/2021 | Japan´s Ministry of Health, Labour and Welfare; Japan´s Ministry of Agriculture, Forestry and Fisheries | OECD, 2022  https://www.mhlw.go.jp/english/; https://www.maff.go.jp/e/ |
| Tiger puffer (*Takifugu rubripes*) | Enhanced (muscle) growth | Approved for commercial sale | Japan/2021 | Japan´s Ministry of Health, Labour and Welfare; Japan´s Ministry of Agriculture, Forestry and Fisheries | OECD, 2022  https://www.mhlw.go.jp/english/; https://www.maff.go.jp/e/ |
| Nile tilapia (*Oreochromis niloticus*) | Enhanced (muscle) growth | Approved for commercial sale | Argentina/2018 | CONABia^[[5]](#footnote-5)^ | Genetic Literacy Project, 2019 |
|  |  | RA^2^ consultation | Brazil/2019 | CTN Bio^4^ | OECD, 2021  http://ctnbio.mctic.gov.br/tecnologias-inovadoras-de-melhoramento-genetico-rn16- |

1. US Food and Drug Administration [↑](#footnote-ref-1)
2. Risk assessment [↑](#footnote-ref-2)
3. Canadian Food Inspection Agency [↑](#footnote-ref-3)
4. National Technical Commission of Biosafety [↑](#footnote-ref-4)
5. National Advisory Commission on Agricultural Biosafety, Argentina [↑](#footnote-ref-5)
