# Supplementary material for: Horizon scanning of potential environmental applications of terrestrial animals, fish, algae and microorganisms produced by genetic modification, including the use of new genomic techniques
Source: Front Genome Ed. 2024 Jun 13;6:1376927. doi: 10.3389/fgeed.2024.1376927 (PMC11208717; doi:10.3389/fgeed.2024.1376927)
Supplement: Supplementary file 5 [file Table4.docx]

Supplementary Material

Supplementary Table 20: Applications of GM algae (BR = basic research, AR = advanced research, MD = market development, m = marine species)

| **Taxon** | **Modified Organism** | **Field of Application** | **Purpose of Development/Intended Trait** | **Level of development** | **Reference** |
| --- | --- | --- | --- | --- | --- |
| Chlorophyta | *Chlamydomonas reinhardtii* | bioremediation | metal absorption (Copper) | BR | Y. Wang et al. (2015) |
| Chlorophyta | *Chlamydomonas reinhardtii* | bioremediation | metal absorption (Nickel) | BR | Zheng et al., 2013 |
| Chlorophyta | *Chlamydomonas reinhardtii* | carbon sequestration | CO_2_ removal efficiency | BR | Asadian et al., 2022 |
| Chlorophyta | *Chlamydomonas reinhardtii* | biofuels | Enhanced triacylglycerol biosynthesis | BR | Deng et al., 2013 |
| Chlorophyta | *Chlamydomonas reinhardtii* | biofuels | Increased lipid content | BR | Ibáñez-Salazar et al., 2014 |
| Chlorophyta | *Chlamydomonas reinhardtii* | biofuels | Fatty acid secretion | BR | Jia et al., 2016 |
| Chlorophyta | *Chlamydomonas reinhardtii* | biofuels | Increase in intracellular lipid content | BR | Jia et al., 2019 |
| Chlorophyta | *Chlamydomonas sp.* | biofuels | Lipid accumulation | BR | Nguyen et al., 2020 |
| Bacillariophyta (diatom) | *Fistulifera solaris* (m) | biofuels | Increased lipid productivity | BR | Osada et al., 2017 |
| Chlorophyta | *Chlamydomonas reinhardtii* | biofuels | modification of the CrACCase gene | BR | Pratami et al., 2022 |
| Ochrophyta | *Nannochloropsis salina* | biofuels | Increased growth | BR | Vikramathithan et al., 2020 |
| Ochrophyta | *Phaeodactylum tricornutum* | biofuels | Increased lipid content | BR | X. Wang et al., 2015 |
| Chlorophyta | Chlamydomonas reinhardtii i | biofuels | light-induced enhanced hydrogen production | BR | Wang et al., 2017 |
| Chlorophyta | Chlamydomonas reinhardtii | biofuels | Improved photobio-H_2_ production | BR | Li et al., 2018 |
| Chlorophyta | *Chlamydomonas reinhardtii* | bioremediation | Herbicide removal (Penoxsulam) | AR | Ismaiel et al., 2019 |
| Chlorophyta | *Chlamydomonas reinhardtii* | bioremediation | Metal removal (Cadmium) | AR | Piña-Olavide et al., 2020 |
| Chlorophyta | *Chlamydomonas reinhardtii* | bioremediation | Cyanide removal | AR | Sobieh et al., 2022 |
| Chlorophyta | *Chlamydomonas reinhardtii* | biocontrol  (human vector control, *Aedes aegypti*) | Silencing of hormone receptor (HR3) to prevent development and molting | AR | Fei et al., 2020 |
| Chlorophyta | *Chlamydomonas reinhardtii* | disease control | Oral vaccine delivery for shrimp cultures (white spot syndrome virus) | AR | Kiataramgul et al., 2020 |
| Chlorophyta | *Chlamydomonas reinhardtii* | disease control | Production of antimicrobial peptide (cecropin B) | AR | Mu et al., 2012 |
| Chlorophyta | *Chlamydomonas reinhardtii* | disease control | Expression of antiviral dsRNA against shrimp virus | AR | Charoonnart et al., 2019 (cited in Sproles et al., 2021) |
| Chlorophyta | *Chlorella* sp. | disease control | Two marine antimicrobial peptides (AMPs) | AR | He et al., 2018 (cited in Sproles et al., 2021) |
| Ochrophyta | *Nannochloropsis oculata* | disease control | bovine lactoferricin (LFB) antibiotic, Red fluorescent protein | AR | Li and Tsai, 2009 (cited in Liang et al., 2020 |
| Ochrophyta | *Nannochloropsis oculata* | feed additive | fish growth hormone (GH) | AR | Chen et al. (2008) cited in Liang et al., 2020 |
| Chlorophyta | *Dunaliella salina* | disease control | white spot syndrome virus VP28 protein | AR | Feng et al., 2014 (cited inSproles et al., 2021) |
| Chlorophyta | *Chlorella sp.* | carbon sequestration | Increased carbon capture and fixation | AR | Lin et al., 2018 |
| Chlorophyta | *Chlamydomonas reinhardtii* | biofuels | Altered fatty acid profile | AR | Ahmad et al., 2015 |
| Chlorophyta | *Chlamydomonas reinhardtii* | biofuels | Increased lipid and fatty acid content | AR | Kim et al., 2019 |
|  | *algae (species n.i.)* | biofuels | Increased photobiological production of hydrogen from water | AR | Lee et al. 2013 |
| Chlorophyta | *Chlamydomonas reinhardtii* | biofuels | Increased lipid production without compromising growth (Sproles et al., 2021; CBAN Update, 2023) | AR | Tan and Lee, 2017 |
| Chlorophyta | *Chlamydomonas reinhardtii* | biofuels | Increased lipid content, esp. C18 and C18:1 | AR | Wang et al., 2018 |
| Ochrophyta | *Phaeodactylum tricornutum* | biofuels | Increased lipid content | AR | Yang et al., 2015 |
| Ochrophyta | *Chaetoceros gracilis* (m) | production/containment | Utilization of phosphite as a sole source of phosphorus | MD | Inoue et al., 2022 |
| Chlorophyta | *Scenedesmus dimorphus, Acutodesmus dimorphus* UTEX 1237 | biofuels | Increase in C14:0 fatty acid synthesis/GFP | MD | Szyjka et al., 2017 |
| Ochrophyta | *Nannochloropsis oceanica* | biofuels | altered fatty acid composition; inability to use nitrate as a nitrogen source | MD | OGTR, 2020 |
| Chlorophyta | *Prototheca morimorfis* | biofuels | Triglyceride and byproducts | MD | OECD, 2021 |
